# The synthetic TRPML1 agonist ML-SA1 rescues Alzheimer-related alterations of the endosomal-autophagic-lysosomal system

**DOI:** 10.1242/jcs.259875

**Published:** 2023-03-21

**Authors:** Aleksandra Somogyi, Emily D. Kirkham, Emyr Lloyd-Evans, Jincy Winston, Nicholas D. Allen, John J. Mackrill, Karen E. Anderson, Phillip T. Hawkins, Sian E. Gardiner, Helen Waller-Evans, Rebecca Sims, Barry Boland, Cora O'Neill

**Affiliations:** ^1^ School of Biochemistry and Cell Biology, BioSciences Institute, University College Cork, T12 YT20 Cork, Ireland; ^2^ Department of Pharmacology and Therapeutics, Western Gateway Building, University College Cork, T12 XF62 Cork, Ireland; ^3^ Cork Neuroscience Centre (CNSC), University College Cork, T12 YT20 Cork, Ireland; ^4^ School of Biosciences, Sir Martin Evans building, Cardiff University, CF10 3AX Cardiff, UK; ^5^ UK Dementia Research Institute, Hadyn Ellis Building, Cardiff University, CF24 4HQ Cardiff, UK; ^6^ Department of Physiology, School of Medicine, University College Cork, T12 YT20 Cork, Ireland; ^7^ The Babraham Institute, Babraham Research Campus, CB22 3AT Cambridge, UK; ^8^ Medicines Discovery Institute, Main Building, Cardiff University, CF10 3AT Cardiff, UK; ^9^ Division of Psychological Medicine and Clinical Neuroscience, Cardiff University, C14 4XN Cardiff, UK

**Keywords:** Alzheimer's disease, TRPML1, Ca^2+^, APOE, PIKfyve, Phosphoinositides

## Abstract

Abnormalities in the endosomal-autophagic-lysosomal (EAL) system are an early event in Alzheimer's disease (AD) pathogenesis. However, the mechanisms underlying these abnormalities are unclear. The transient receptor potential channel mucolipin 1(TRPML1, also known as MCOLN1), a vital endosomal-lysosomal Ca^2+^ channel whose loss of function leads to neurodegeneration, has not been investigated with respect to EAL pathogenesis in late-onset AD (LOAD). Here, we identify pathological hallmarks of TRPML1 dysregulation in LOAD neurons, including increased perinuclear clustering and vacuolation of endolysosomes. We reveal that induced pluripotent stem cell (iPSC)-derived human cortical neurons expressing APOE ε4, the strongest genetic risk factor for LOAD, have significantly diminished TRPML1-induced endolysosomal Ca^2+^ release. Furthermore, we found that blocking TRPML1 function in primary neurons by depleting the TRPML1 agonist PI(3,5)P_2_ via PIKfyve inhibition, recreated multiple features of EAL neuropathology evident in LOAD. This included increased endolysosomal Ca^2+^ content, enlargement and perinuclear clustering of endolysosomes, autophagic vesicle accumulation and early endosomal enlargement. Strikingly, these AD-like neuronal EAL defects were rescued by TRPML1 reactivation using its synthetic agonist ML-SA1. These findings implicate defects in TRPML1 in LOAD EAL pathogenesis and present TRPML1 as a potential therapeutic target.

## Teaser

**Summary:** TRPML1 defects accompany abnormalities in the endolysosomal system in Alzheimer's disease and APOE-ε4 iPSC-derived neurons, which are recapitulated by neuronal PIKfyve inhibition and remediated by the TRPML1 agonist ML-SA1.

## INTRODUCTION

Increasing research evidence implicates defects in the endosomal-autophagic-lysosomal (EAL) system as a very early event in the genesis and progression of Alzheimer's disease (AD) pathology, including the buildup of amyloid-β (Aβ) and tau, and synaptic pathogenesis (Lie and Nixon, 2019; Nixon, 2017, 2020; Van Acker et al., 2019; van Weering and Scheper, 2019; Whyte et al., 2017). Furthermore, studies indicate that expression of the APOE ε4-encoding variant of apolipoprotein E (*APOE*) (Lin et al., 2018; Nuriel et al., 2017) is the greatest genetic risk factor for late onset AD (LOAD), which, along with many other LOAD risk genes and genes causing familial AD (Hung and Livesey, 2018; Kwart et al., 2019), converge to alter EAL function (Gao et al., 2018; Karch and Goate, 2015; Pimenova et al., 2018; Van Acker et al., 2019). However, it remains unclear which nodes of the EAL system are primarily affected in AD and, hence, could be targeted therapeutically to remediate AD pathogenesis.

The transient receptor potential channel mucolipin 1 (TRPML1; also known as MCOLN1) is a non-selective cation channel that can transport Ca^2+^, Fe^2+^ and Zn^2+^ (Dong et al., 2008; Kiselyov et al., 2011). The primary function of TRPML1 is to induce Ca^2+^ release from endolysosomal compartments, a vital process for endolysosomal function (Di Paola et al., 2018; Venkatachalam et al., 2015; Waller-Evans and Lloyd-Evans, 2015). TRPML1 is highly expressed in the brain (Samie et al., 2009) and, although not investigated with respect to EAL pathogenesis in LOAD, regulates many EAL functions known to be impaired in AD neurons. The functions regulated by TRPML1 include: maturation of late endosomes to lysosomes; endolysosomal trafficking; nutrient sensing and adaptation; positioning, exocytosis, fission, clearance and reformation of lysosomes; autophagy, phagocytosis and clearance of aggregated proteins and pathogens (reviewed in Di Paola and Medina, 2019; Di Paola et al., 2018; Huang et al., 2020; Kendall and Holian, 2021; Li et al., 2019). Loss-of-function mutations in the human TRPML1 gene *MCOLN1* cause mucolipidosis type IV (MLIV) (Bargal et al., 2001; Bassi et al., 2000; Slaugenhaupt, 2002; Sun et al., 2000), a rare recessive lysosomal storage disorder (LSD). MLIV is characterized by neurodegeneration, psychomotor impairment, ophthalmologic and gut defects (Boudewyn and Walkley, 2019). MLIV patient cells show multiple EAL abnormalities including defective endolysosomal trafficking, vacuolation, altered positioning of the endolysosomal compartment, compromised maturation of lysosomes, dysregulated pH and autophagic defects (for a review, see Boudewyn and Walkley, 2019; Cheng et al., 2010; Puertollano and Kiselyov, 2009).

The link between endolysosomal TRPML1 dysfunction and neurodegenerative disease is further supported by findings that TRPML1-mediated Ca^2+^ release is impaired in other LSDs, including Niemann–Pick C (NPC) disease (Lloyd-Evans and Platt, 2011; Shen et al., 2012). NPC disease shares the pathological features of tau and Aβ accumulation with AD (Lloyd-Evans et al., 2008). Furthermore, although not yet examined in LOAD, alterations in TRPML1 function are associated with deletion of the presenilin-1 (PS-1; also known as *PSEN1*) gene, which causes familial AD (FAD) (Lee et al., 2015, 2010; Lie et al., 2022). TRPML1 function is implicated in autophagic clearance of Aβ and tau in AD by being the primary activator of transcription factor EB (TFEB), the major regulator of lysosomal biogenesis and autophagy (Medina et al., 2015; Napolitano and Ballabio, 2016; Zhang et al., 2016). TFEB activation promotes clearance of Aβ and tau pathology in preclinical models of AD (Akwa et al., 2023; Martini-Stoica et al., 2018; Polito et al., 2014; Song et al., 2020; Xiao et al., 2015; Xu et al., 2020), where TRPML1 activation has been shown to be essential for TFEB-mediated clearance of tau-induced pathology (Xu et al., 2020). Recent studies show that TRPML1-dependent lysosomal Ca^2+^ release regulates dendritic lysosomal trafficking and hippocampal neuronal function (Sun et al., 2022).

TRPML1 is one of the few ion channels gated by phosphoinositide (PI) lipids, with PI(3,5)P_2_ being the primary and only identified endogenous agonist of TRPML1 (Dong et al., 2010; Zhang et al., 2012), and PI(4,5)P_2_ the TRPML1 antagonist (De Leo et al., 2016; Feng et al., 2014a; Zhang et al., 2012). PI metabolism is central to effective vesicular trafficking in the EAL system (Balla, 2013; Di Paolo and De Camilli, 2006) and attention has been drawn to defects in PI metabolism in the AD brain (Morel et al., 2013; Stokes and Hawthorne, 1987; Zhu et al., 2015) and as a target of LOAD risk genes (reviewed in Raghu et al., 2019). Another interesting feature of this system is that PI(3,5)P_2_, the TRPML1 agonist, is synthesized exclusively by the PIKfyve kinase complex (McCartney et al., 2014). In concordance, inhibition of the PIKfyve complex recreates endolysosomal defects similar to those in MLIV, typified by vacuolation of endolysosomal compartments (Bissig et al., 2017; Chen et al., 2017; Choy et al., 2018; Dong et al., 2010; Ikonomov et al., 2002, 2001; Jefferies et al., 2008; Kim et al., 2014; Martin et al., 2013; McCartney et al., 2014). Furthermore, as with TRPML1, mutations in the human PIKfyve complex, namely the FIG4 and Vac14 components, lead to neurodegeneration – in this case, including amyotrophic lateral sclerosis (ALS), Charcot–Marie–Tooth disease, and Yunis–Varon syndrome (Campeau et al., 2013; Chow et al., 2009; Chow et al., 2007; Lines et al., 2017; Nicholson et al., 2011; Zhang et al., 2008).

Interestingly, recent studies have shown that loss of PIKfyve activity drives spongiform neurodegeneration and neuronal vacuolation in prion disease, which can be rescued by PI(3,5)P_2_ supplementation (Lakkaraju et al., 2021). Conversely, other publications show that PIKfyve inhibition reduces the transport of tau and α-synuclein from early to late endolysosomes *in vitro*, preventing their fibrillization, which is thereby considered as neuroprotective (See et al., 2021 preprint; Soares et al., 2021). Together, this draws attention to investigating the potential mechanistic link between PIKfyve activity and TRPML1 function in the context of EAL neuropathogenesis in LOAD and other neurodegenerative diseases. It has been suggested that activation of TRPML1 might represent a protective strategy against EAL defects in LOAD (Hui et al., 2019). Importantly, synthetic small-molecule compounds, including mucolipin synthetic agonist 1 (ML-SA1) have been developed as PI(3,5)P_2_-independent specific TRPML1 agonists (Fine et al., 2020; Grimm et al., 2010; Shen et al., 2012). ML-SA1 has been shown to protect against Alzheimer's-like Aβ pathology in other neurological conditions, particularly HIV-1-associated neurocognitive disorders (Bae et al., 2014; Hui et al., 2019, 2021). Furthermore, ML-SA1 protects against endolysosomal pathology caused by FIG4 deficiency (Zou et al., 2015). However, whether these TRPML1 agonists can protect against potential EAL pathogenesis due to direct PIKfyve inhibition in neurons is unknown.

In this study, we hypothesized that dysregulation of TRPML1-mediated endolysosomal function is an underlying component of EAL neuropathogenesis in LOAD. Therefore, we aimed to determine whether indicators of TRPML1 function were altered in LOAD patient brains and in induced pluripotent stem cell (iPSC)-derived neurons expressing APOE ε4 compared to cells with other APOE isoforms. We further investigated whether it was possible to model key AD-related EAL phenotypes in primary neurons by inactivating TRPML1 via inhibition of PI(3,5)P_2_ production, using the PIKfyve inhibitor YM201636. Finally, we investigated whether targeting TRPML1 with ML-SA1 in primary neurons could provide a novel approach to remediate AD-related EAL defects induced by PIKfyve inhibition.

## RESULTS

### Endolysosomal neuropathology in the AD brain and altered phosphoinositide dynamics indicate abnormalities in TRPML1 function

**Fig. 1.**
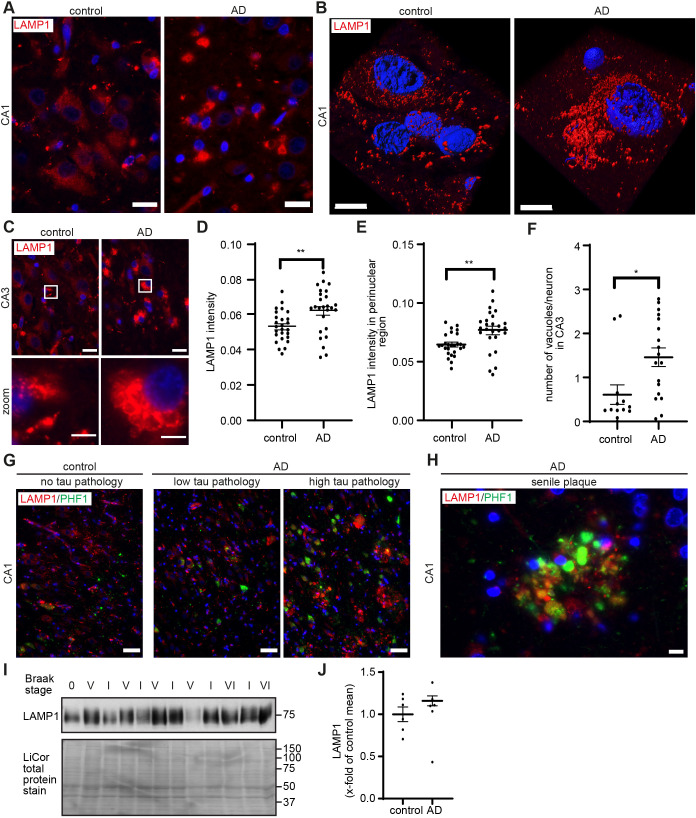
**Increased levels and altered subcellular distribution of LAMP1-positive endolysosomes in the AD brain.** (A,C) Representative images showing the accumulation and swelling of LAMP1-positive vesicles in cells of the CA1 (A) and CA3 (C) regions of hippocampal sections of AD (*n*=10) and control cases (*n*=10). Scale bars: 20 µm. Detailed sections of single neurons are shown in bottom panel of C. Scale bars: 5 µm. (B) Surface-rendered 3D reconstruction of LAMP1-positive vesicles in a morphologically identified CA1 pyramidal neuron of an AD patient and control. Scale bars: 10 µm. (D,E) Quantification of LAMP1 intensity in the CA1 region (D) and specifically in the perinuclear area (E) from *n*=6 AD cases and *n*=8 control cases with 2–5 representative images analysed for each case, together analysing *n*=26 control images and *n*=26 AD images. (F) Quantification of the number of enlarged LAMP1 immunoreactive vesicles per neuron in the CA3 region. (G) Representative images showing increased LAMP1 immunoreactivity in cells of the CA1 region accumulating PHF-1 immunoreactive tau in AD patients (*n*=10, right) and control (*n*=10, left), and areas of AD CA1 with little PHF1 immunoreactive tau (middle). (H) Representative image of LAMP1 localisation to senile plaques decorated with PHF-1 immunoreactive tau (*n*=10 AD cases). (I) Western immunoblot analysis of temporal cortex membrane fractions (*n*=6) showing a trend towards increased LAMP1 levels in AD patients compared with controls (n.s.). LiCor total protein stain was used to ensure equal loading. (J) Quantification of LAMP1 immunoblot data. Data are expressed as mean±s.e.m. *P<0.05; **P<0.01 (unpaired two-tailed Student's *t*-test).

TRPML1 function and PI dynamics closely relate to endolysosomal integrity (Cao et al., 2017; Zhang et al., 2012). We thus first interrogated endolysosomal integrity in post-mortem AD brain tissue using an antibody against the endogenous endolysosomal marker LAMP1, performing immunofluorescence microscopy of post-mortem hippocampal sections from AD patients (*n*=10) and matched controls (*n*=10) (Table 1). Our results demonstrated an increase in LAMP1-positive endolysosomes, which was particularly evident in the perinuclear region of hippocampal cells in the CA1 and CA3 regions, in AD cases compared to in the matched controls (Fig. 1A–C). A 3D reconstruction of LAMP1 immunoreactivity and positioning in a morphologically identifiable CA1 pyramidal neuron in AD compared to control pyramidal neurons is shown in Fig. 1B. Quantification, using CellProfiler, revealed that LAMP1 intensity was significantly increased in the perinuclear area of cells in the CA1 region in AD compared to in control cases (Fig. 1D,E).

**Table 1.**
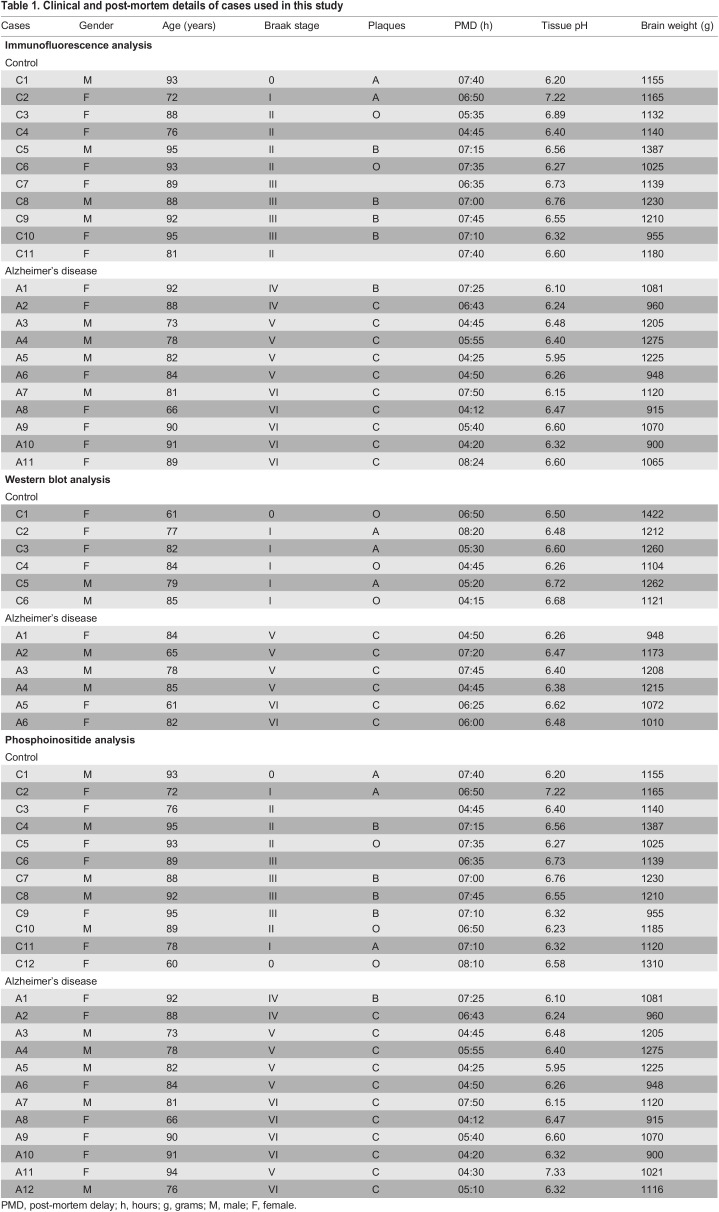
Clinical and post-mortem details of cases used in this study

We performed double immunofluorescence with LAMP1 and the astrocytic marker GFAP to investigate the cellular localisation of LAMP1 pathogenesis in the AD brain. Here, our results showed that increased levels of LAMP1-positive endolysosomes were observed in the perinuclear regions of GFAP-negative pyramidal neurons in the AD hippocampus (Fig. S1A). GFAP-labelled astrocytes with increased levels of LAMP1 immunoreactivity in perinuclear regions were also identified in the AD hippocampus (Fig. S1A).

Deletion of TRPML1 in MLIV and inhibition of the synthesis of the TRPML1 agonist PI(3,5)P_2_ induce endolysosomal defects including perinuclear clustering, as described above, as well as enlargement of endolysosomal vesicles (Dong et al., 2010; McCartney et al., 2014; Zhang et al., 2012). Notably, vacuolisation of LAMP1-positive vesicles was observed in cells with the morphology of neurons in the CA3 region in AD cases (Fig. 1C). Quantification using ImageJ revealed a significant increase in the number of enlarged LAMP1-positive vesicles in the CA3 region in AD compared to in control cases (Fig. 1F). Vacuoles were defined as large membrane-bound vesicular structures immunoreactive to LAMP1. These vacuoles were larger than vesicles (∼4–10 µm diameter). In granulovacuolar degeneration (GVD), described to occur in the AD brain (Funk et al., 2011; Kohler, 2016; Thal et al., 2011), vacuoles are described to be 3–5 µm and to have a dense central core or central granule of 0.5–1.5 µm, which was not evident in the vacuoles we show. We attempted to determine whether these vacuoles were similar to those observed in GVD, using casein kinase-1δ (CK-1δ), a marker for the granulovacuolar granule (Funk et al., 2011) and double immunofluorescence with LAMP1. However, immunofluorescence analysis with CK-1δ antibody did not reveal clear granulovacuolar granule staining (data not shown).

In the AD cases, increased LAMP1 immunoreactivity was greater in areas where many cells accumulated PHF-1 immunoreactive tau (Fig. 1G, right) and was strongly enriched around senile plaques (Fig. 1H). The observation that LAMP1 immunoreactivity is enriched in plaques has been described in the AD brain (Barrachina et al., 2006) and in preclinical mouse AD models (Condello et al., 2011; Gowrishankar et al., 2015; Kandalepas et al., 2013). Control and AD brain material was staged for disease severity (Braak staging 0–VI, see Materials and Methods). Alterations in the levels of LAMP1 and endolysosomal enlargement were specific to AD cases irrespective of their Braak stage. However, western immunoblot analysis (Fig. 1I) and quantification (Fig. 1J) of temporal cortex membrane fractions prepared from AD and control brain showed only a non-significant trend towards an increase in LAMP1 levels in many AD samples compared with control levels, which is in line with previous literature (Bordi et al., 2016), and points rather to a shift in localization resulting in regional accumulation of lysosomes in the perinuclear region.

**Fig. 2.**
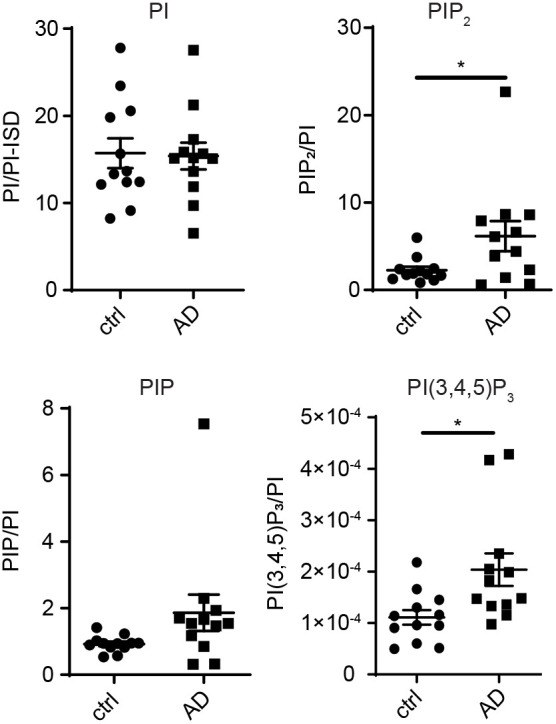
**Levels of phosphoinositides with the potential to regulate TRPML1 are altered in the temporal cortex of AD patients.** HPLC-MS analysis of PI per PI-internal standard (PI/PI-ISD), as described in the Materials and Methods. Levels of total PIP, PIP_2_ and PI(3,4,5)P_3_ in mid temporal cortex tissue of AD (*n* =12) and control (*n*=12) groups. Levels of total PIP_3_ [PI(3,4,5)P_3_] and total PIP_2_ [PI(4,5)P_2_, PI(3,4)P_2_ and PI(3,5)P_2_], were significantly increased (*P<0.05, unpaired two-tailed Student's *t*-test) in AD cases compared to controls. Regio-isomer classification showed that increased total PIP_2_ levels in AD cases represented PI(4,5)P_2_ (data not shown). Data are expressed as mean±s.e.m.

As TRPML1 is a PI-gated ion channel (Dong et al., 2010; Feng et al., 2014a; Fine et al., 2018; Hille et al., 2015), which relies heavily on effective PI dynamics, we thought it important to determine whether levels of all measurable PIs were altered in the AD brain. We quantified individual and total PI levels in brain tissue of the AD and age-matched control groups using advanced mass spectrometry approaches (Kielkowska et al., 2014). Results demonstrated that levels of total PIP_3_ [PI(3,4,5)P_3_] and total PIP_2_ [regioisomers PI(4,5)P_2_, PI(3,4)P_2_ and PI(3,5)P_2_] were significantly increased (*P*<0.05) in the mid temporal cortex of AD patients (Braak IV–VI, *n*=12) compared to age-matched controls (Braak 0–III, *n*=12) (Table 1; Fig. 2). No significant differences were detected in PI or total PIP [PI3P, PI4P and PI5P] levels when comparing AD and control groups (Fig. 2). There was no correlation between PI levels and post-mortem delay in these samples. Regio-isomer classification of the primary (> 75%) lipid species (stearoyl/arachidonoyl, ‘C38:4’) showed that increased PIP_2_ levels represented PI(4,5)P_2_, as no PI(3,4)P_2_ levels were detected (data not shown). Unfortunately, PI(3,5)P_2_, the major agonist of TRPML1, cannot be distinguished yet using mass spectrometry methodology as the abundance of this lipid species is extremely low (Michell et al., 2006). Significantly, PIP_3,_ whose levels we show to be increased in the AD group, is the major activator of Akt protein kinases, which are known to be hyper-activated in AD neurons (Griffin et al., 2005; Moloney et al., 2010) and which are a primary regulator of EAL dynamics via the mTOR signalling axis (Boland et al., 2018). Taken together, the PI changes observed in AD brains reflect significant defects in overall PI network integrity that have the potential to impact TRPML1 and endolysosomal function in AD.

We performed western immunoblot and immunofluorescence analysis with TRPML1 antibodies in control and AD brain. TRPML1 antibodies have been criticised due to possible non-specificity and we found variability in antigenicity and immunoblot profiles between TRPML1 antibody batches (Fig. S1B,C). Thus, we were unable to make a definitive conclusion on whether the levels or subcellular localisation of TRPML1 were altered in the AD brain compared to matched controls. Nonetheless, we have included representative immunoblots and immunofluorescence analysis of TRPML1 in control and AD cases in Fig. S1B–D.

### Lysosomal Ca^2+^ levels are increased and TRPML1 activity is decreased in homozygous APOE ε4 neurons

**Fig. 3.**
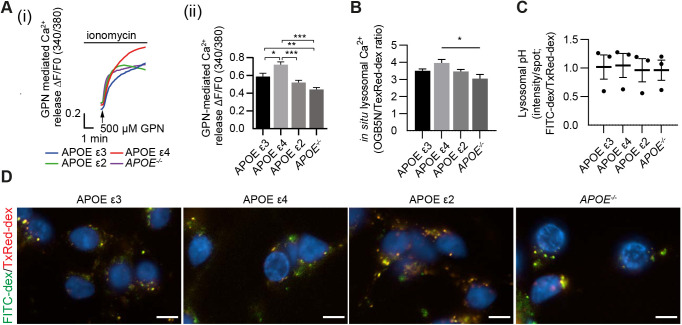
**Increased lysosomal Ca^2+^ levels in a neuronal LOAD iPSC model.** (A) APOE ε3, APOE ε4, APOE ε2 or *APOE*^−/−^ iPSC neurons were loaded with Fura2-AM and treated with ionomycin followed by GPN to release lysosomal Ca^2+^. Representative traces (i) and quantification (ii) of GPN-induced Ca^2+^ release following ionomycin pre-treatment. (B) Quantification of *in situ* Ca^2+^ levels as fluorescence ratio between the Ca^2+^-sensitive OGB (0.5 mg/ml) and Ca^2+^-insensitive Texas Red–Dextran (0.1 mg/ml) as loading control in *n*=5 biological replicates, *n*=1 technical replicate per *APOE* isoform. (C,D) Quantification (C) and representative images (D) of APOE ε3, APOE ε4, APOE ε2 or *APOE*^−/−^ iPSC neurons loaded with pH-sensitive FITC–dextran (0.5 mg/ml) and pH-insensitive Texas Red–dextran (0.25 mg/ml), as loading control, showed no difference in lysosomal pH. Scale bars: 10 µm.

Enlargement of the endolysosomal system has also been described in APOE ε4 modified cell systems *in vivo* (Nuriel et al., 2017; Xian et al., 2018), reflecting our findings in neurons in the LOAD brain. A major functional consequence of defective TRPML1 function is impaired Ca^2+^ efflux from late endolysosomes. TRPML1 activity is not measurable in post-mortem brain tissue. We thus applied a more dynamic physiological measurement of endolysosomal Ca^2+^ in an *APOE*-modified AD neuronal system where we differentiated human iPSC-derived neurons from cells expressing isogenic APOE ε3, APOE ε4 and APOE ε2, and *APOE^−/−^* cells (Schmid et al., 2019). Measurement of endolysosomal Ca^2+^ was obtained by imaging Fura2-AM-labelled iPSC neurons that were first treated with the Ca^2+^ ionophore ionomycin (2 µM), which induces Ca^2+^ release from the endoplasmic reticulum (ER), followed by the lysosomal membrane disrupting agent, glycyl-L-phenylalanine 2-naphthylamide (GPN, 500 µM) to release endolysosomal Ca^2+^ into the cytosol (Bergling et al., 1998; Sage et al., 2011). Hence, the resulting elevation in fluorescence of the Ca^2+^ probe allows for an estimation of the lysosomal Ca^2+^ content. Using this experimental paradigm, we found that neurons expressing APOE ε4, the greatest genetic risk factor for LOAD, had significantly higher levels of lysosomal Ca^2+^ compared with APOE ε3 (*P*=0.0102), APOE ε2 (*P*<0.0001) and *APOE*^−/−^ (*P<*0.0001) neurons (Fig. 3A).

We performed further experiments to measure lysosomal Ca^2+^ *in situ* with Oregon Green-conjugated BAPTA 5N (OGB) and Texas Red-conjugated dextran as a loading control (Fig. 3B). Although not significant compared with APOE ε3 and APOE ε2, the trend seen in these data further confirms that lysosomal Ca^2+^ is indeed elevated in APOE ε4-expressing iPSC neurons (*P*=0.0116 versus *APOE*^−/−^). As Ca^2+^ accumulation itself and the *K*_D_ of OGB are affected by endolysosomal vacuolar pH (Gerasimenko et al., 1998), we measured endolysosomal pH, using a pH-sensitive FITC–dextran alongside Texas Red–dextran as loading control. Our results show that endolysosomal pH is not changed in any of the *APOE* isoform-expressing cortical neurons (Fig. 3C,D). This removes any concerns about possible alterations in vacuolar pH in these neurons. It also gives confidence that GPN in these neurons is being hydrolysed correctly and that the Ca^2+^ buffering capacity of OGB was unchanged.

**Fig. 4.**
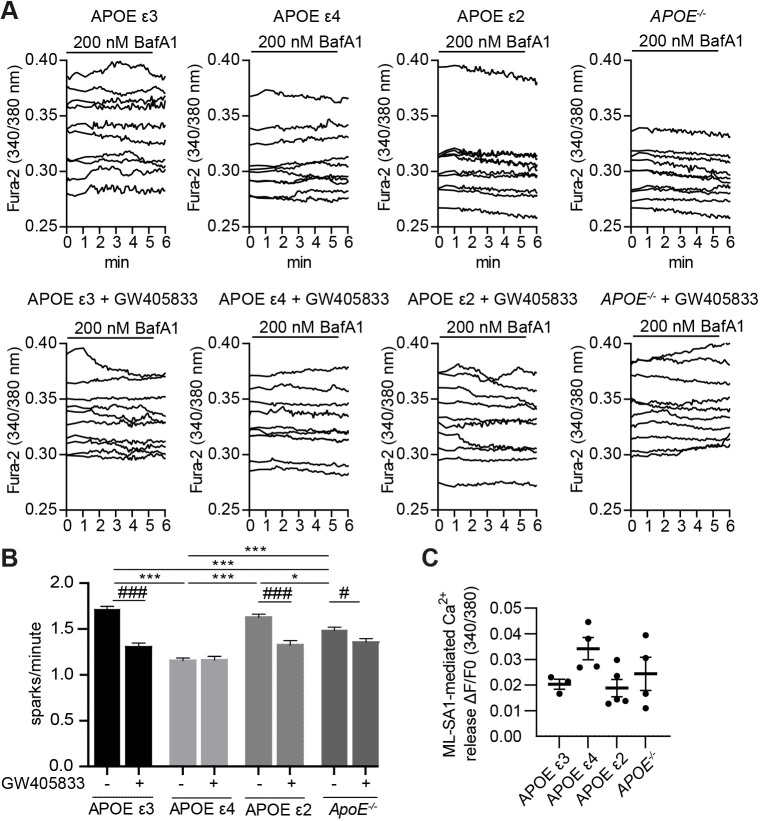
**Decreased TRPML1-mediated lysosomal Ca^2+^ release in a neuronal LOAD iPSC model.** (A,B) APOE ε3, APOE ε4, APOE ε2 or *APOE*^−/−^ iPSC neurons were loaded with Fura2-AM and treated with the IP_3_ receptor antagonist xestospongin C to block Ca^2+^ efflux from the ER, followed by BafA1 to mimic age-related mild deacidification. TRPML1 activity was assessed by counting spontaneous sparks of Ca^2+^ release during a 5-min period in the presence [APOE ε3 (*n*=11, 235 traces), APOE ε4 (*n*=12, 243 traces)*,* APOE ε2 (*n*=10, 226 traces), *APOE*^−/−^ (*n*=11, 257 traces)] or absence [APOE ϵ3 (*n*=6, 90 traces)*,* APOE ϵ4 (*n*=6, 101 traces)*,* APOE ϵ2 (*n*=6, 130 traces), *APOE*^−/−^ (*n*=7, 163 traces)] of the TRPML1 inhibitor GW405833 (10 µM). The response of representative cells is depicted and expressed as the 340/380 nm ratio of Fura2-AM florescence. Representative traces (A) and quantification (B) of spark number/minute induced by BafA1. Significance levels were calculated between all samples without GW405833 to detect *APOE* isoform-specific alterations in TRPML1 response (*P<0.05; **P<0.01; ***P<0.001, one-way ANOVA followed by Bonferroni post-hoc test) and for each *APOE* isoform between GW405833-treated and untreated sample to assess TRPML1 contribution (^#^*P*<0.05; ^###^*P*<0.001, unpaired two-tailed Student's *t*-test). (C) Quantification of full physiological cellular Ca^2+^ release after addition of 10 µM ML-SA1 including, but not limited to lysosomal Ca^2+^ stores in *n*=3 biological replicates, *n*=1 technical replicate per *APOE* isoform. Data are expressed as mean±s.e.m.

To assess endogenous TRPML1 activity in finer detail, these neurons were treated with low concentrations (200 nM) of bafilomycin A1 (BafA1), which causes a mild increase in lysosomal pH (Yoshimori et al., 1991), indicated to increase TRPML1-mediated Ca^2+^ release (Lee et al., 2015; Li et al., 2017). Following, BafA1 addition, the number of spontaneous Ca^2+^ sparks was counted during a 5-min period. Results demonstrate that APOE ε3, APOE ε2 and *APOE*^−/−^ neurons had an average of 1.71±0.03, 1.63±0.03 and 1.49±0.04 spontaneous Ca^2+^ sparks/min, respectively (mean±s.e.m.). In contrast, APOE ε4 neurons had a significantly lower number of spontaneous Ca^2+^ sparks/min (1.16±0.03, *P*<0.0001) (Fig. 4A,B). To determine whether these Ca^2+^ sparks were TRPML1 mediated, neurons were pre-treated with the TRPML1 inhibitor GW405833, a close analogue of ML-SI1 (Rautenberg et al., 2022). Upon TRPML1 inhibition, the number of spontaneous Ca^2+^ sparks were significantly reduced (*P*<0.001, Fig. 4A,B) to 1.30±0.04 and 1.33±0.05 sparks/min in APOE ε3 and APOE ε2 neurons, respectively (Fig. 4A,B) and to 1.36±0.04 sparks in *APOE*^−/−^ neurons (*P*=0.016). In contrast, APOE ε4 neurons showed no change in the number of spontaneous sparks/min upon TRPML1 inhibition (Fig. 4A,B).

Treatment with the synthetic TRPML1 agonist ML-SA1, which locks the endolysosomal TRPML1 channel non-physiologically in an open conformation (Feng et al., 2014b), showed that ML-SA1 could induce TRPML1-mediated Ca^2+^ release regardless of the *APOE* isoform expressed (Fig. 4C). The ML-SA1-induced Ca^2+^ release was not significantly different when comparing *APOE* isoforms, although there was a trend towards increased Ca^2+^ release in the APOE ε4 cortical neurons (Fig. 4C). This increase is most likely due to an ER compensatory effect, as ER Ca^2+^ release was not blocked in these experiments, and the fact that ML-SA1 locks the channel in an open conformation.

The cation selectivity for TRPML1 channels has been described to include Ca^2+^, Fe^2+^ and Zn^2+^ (Dong et al., 2008; Kiselyov et al., 2011). We performed experiments using the FluoZin3(AM) probe to detect Zn^2+^ accumulation in endolysosomes. Our results show that there were no significant differences in Zn^2+^ levels between the cortical neurons expressing the different *APOE* isoforms (Fig. S2A–E). This is not surprising, as Zn^2+^ accumulation in cells with a TRPML1 defect is only observed when the cells are grown in 100 µM extracellular Zn^2+^ (Minckley et al., 2019). We did not investigate this, as this concentration of ZnCl_2_ is toxic to cells. The Fura-2 fluorophore can detect Zn^2+^ in theoretical experimental conditions when Zn^2+^ concentrations are high (Martin et al., 2006). These Zn^2+^ concentrations are not attained in the cellular context (Bozym et al., 2010, 2006), where Fura-2 has been shown to be unable to respond to the small physiological changes in cellular Zn^2+^ concentrations (Krezel and Maret, 2006). TPEN can be used as a cell permeant Zn^2+^ chelator. However, we did not employ TPEN in these studies, as it also chelates Ca^2+^ (Morgan et al., 2012).

Notably, increased cathepsin D levels have been described previously in AD neurons (Cataldo et al., 1995, 1991). We used BODIPY–pepstatin as an indicator of cathepsin D activity (Chen et al., 2000). Our results show BODIPY-pepstatin fluorescence was altered in APOE ε4 neurons, with significantly increased BODIPY-pepstatin total spot fluorescence (*P*=0.0021 versus APOE ε2, *P*=0.0153 versus *APOE*^−/−^) and spot area (*P*=0.0134 versus APOE ε2) in *APOE* ε4 neurons compared to other *APOE* isoforms (Fig. S2F–H). There was also a trend towards fewer BODIPY–pepstatin fluorescent spots per cell in the *APOE* ε4 neurons, but this was not significant (Fig. S2I). These data indicate that there are increased levels of active cathepsin D in APOE ε4 neurons, further indicating, albeit indirectly, that lysosomes are not de-acidified in APOE ε4 neurons.

Together, our results indicate that APOE ε4 neurons, which model genetic risk for LOAD, have significantly higher levels of endolysosomal Ca^2+^ and are unable to release Ca^2+^ via TRPML1 in response to induced mild deacidification of endolysosomal compartments. These defects in the ability of TRPML1 to release Ca^2+^ in APOE ε4 neurons occur in the absence of any alteration in endolysosomal pH or endolysosomal Zn^2+^ levels.

### Inhibition of PIKfyve causes AD-like increases in endolysosomal Ca^2+^ content, which are rescued by the TRPML1 agonist ML-SA1

**Fig. 5.**
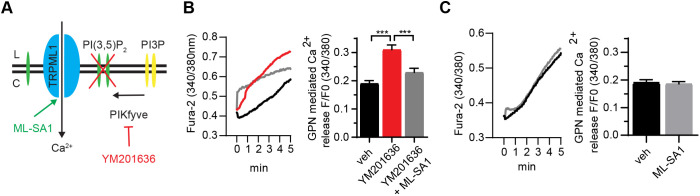
**ML-SA1 rescues YM201636-mediated Ca^2+^ accumulation in rat primary cortical neurons.** (A) Scheme of TRPML1 inactivation by PIKfyve inhibition and reactivation by ML-SA1. (B) Lysosomal Ca^2+^ was measured in neurons loaded with Fura2-AM, using 500 µM GPN to release lysosomal Ca^2+^ following a 2 µM ionomycin pre-treatment to clamp all other intracellular Ca^2+^ stores, in rat primary cortical neurons pre-treated with 4 µM YM201636 (red, *n*=6, 72 traces) for 24 h when compared to vehicle (veh, black, *n*=7, 118 traces) only. Co-treatment with 50 µM ML-SA1 (grey, *n*=6, 77 traces) restored the lysosomal Ca^2+^ pool. Representative trace (left) and quantification (right). (C) Rat primary cortical neurons were pre-treated with either 50 µM ML-SA1 (grey, *n*=3, 388 traces) or vehicle (black, *n*=3, 381 traces) for 24 h. Lysosomal Ca^2+^ content was measured as in B, but no change was detected. ***P<0.001 (one-way ANOVA, followed by Bonferroni post-hoc test). Data are expressed as mean±s.e.m.

PI(3,5)P_2_ is currently the only known endogenous agonist of TRPML1 (Dong et al., 2010; Zhang et al., 2012). Thus, inhibition of PIKfyve, the unique PI(3,5)P_2_-synthesising enzyme complex, by the pharmacological inhibitors YM201636 (Jefferies et al., 2008) and apilimod (Cai et al., 2013) deprives TRPML1 of its agonist and replicates many endolysosomal defects caused by loss of TRPML1 function in non-neuronal systems (Cai et al., 2013; Dong et al., 2010; Jefferies et al., 2008; McCartney et al., 2014). Having described TRPML1-related endolysosomal defects in AD neurons and diminished TRPML1 activity in APOE ε4-expressing iPSC-derived neurons, we were next interested to determine whether these TRPML1 defects could be replicated by PIKfyve inhibition in primary neurons. Here, it is possible to link the effects of PIKfyve inhibition specifically to TRPML1 function within neurons, by investigating whether the small synthetic TRPML1 agonist ML-SA1 protects against any EAL phenotypes induced by PIKfyve inhibition. This was of major interest, as it has not been investigated previously, and allows evaluation of the therapeutic potential of ML-SA1 and thus TRPML1 activation, to protect against AD-like endolysosomal phenotypes in a neuronal model system (Fig. 5A).

Firstly, we treated primary rat cortical neurons with YM201636. Endolysosomal Ca^2+^ content was measured in a similar manner to that in human *APOE*-modified neurons – modifications of the protocol are described in the Materials and Methods and included differing Fura-2 concentrations due to the lysosomal Ca^2+^ release levels in rat primary cortical neurons and lesser sensitivity of the camera used. Our results show that endolysosomal Ca^2+^ levels were significantly increased (*P*<0.0001) in the presence of 4 µM YM201636 for 6 h (1.62±0.10 fold, data not shown; mean±s.e.m.) and 24 h (1.63±0.07 fold), respectively (Fig. 5B). Notably, although scale and effect size are different, this pharmacological inhibition of PIKfyve in rat primary neurons replicates the increased endolysosomal Ca^2+^ content we detected in APOE ε4 neurons. Furthermore, when these neurons were co-treated with ML-SA1 (50 µM), the accumulated Ca^2+^ was released from the lysosomes and the size of lysosomal Ca^2+^ stores were restored to the levels of vehicle-treated cells (*P*=0.0003) (Fig. 5B). ML-SA1 treatment alone did not deplete lysosomal Ca^2+^ stores within the time frames used in this study (Fig. 5C). Together, these results indicate that therapeutic activation of TRPML1 has the potential to protect against increased lysosomal Ca^2+^ levels in primary neurons such as those evident in APOE ε4 LOAD model systems.

### Inhibition of PIKfyve replicates AD-like perinuclear accumulation and vacuolation of endolysosomal compartments, which are rescued by the TRPML1 agonist ML-SA1

**Fig. 6.**
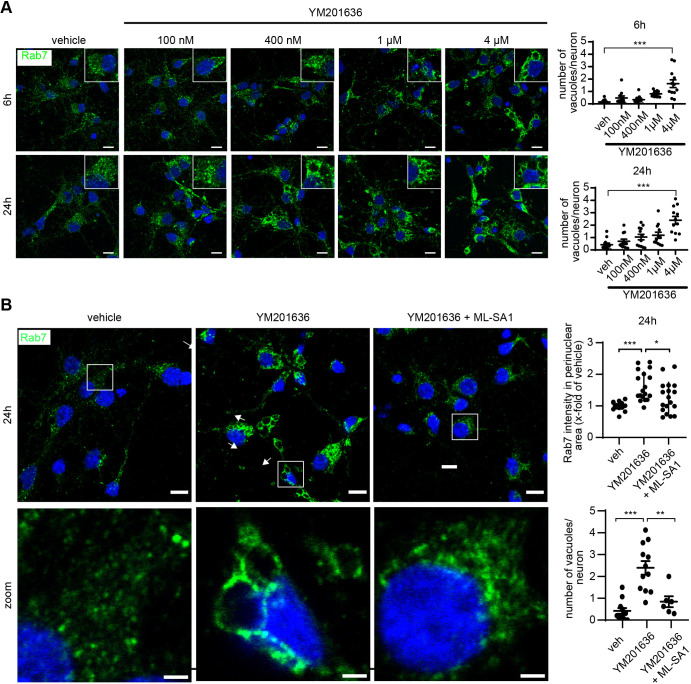
**Enlargement of Rab7-positive endolysosomes is dose dependent for YM201636 and can be rescued by ML-SA1 co-treatment.** (A) Representative images (left) and quantification (right) showing that depletion of PI(3,5)P_2_ using 100 nM–4 µM YM201636 in rat primary cortical neurons led to a dose-dependent increase in the size and intensity of Rab7-positive late endolysosomes. (B) Representative confocal images (left, top row), zoom (left, bottom row) and quantification (right) showing endolysosomal vacuolation and perinuclear accumulation of Rab7-positive vesicles, in rat primary cortical neurons after PIKfyve inhibition using 4 µM YM201636, which is restored by co-treatment with 50 µM ML-SA1. Quantitative data is based on four separate experiments with three images for each condition from two separate coverslips. veh, vehicle. Scale bars: 10 µm (main images) and 2 µm (magnifications). *P<0.05; **P<0.01; ***P<0.001 (one-way ANOVA, followed by Bonferroni post-hoc test). Data are expressed as mean±s.e.m.

Next, we determined whether the perinuclear accumulation and vacuolation of endolysosomes we describe in AD neurons could be replicated by PIKfyve inhibition, and whether this could be rescued by activation of TRPML1 with ML-SA1. Here, we employed the endolysosomal marker Rab7 (Ginsberg et al., 2010), which performed better as an endolysosomal marker in primary neurons when compared to LAMP1. Furthermore, Rab7 is critical for effective late endolysosomal function, including lysosomal biogenesis and positioning in the perinuclear region (for review see Guerra and Bucci, 2016). Our results revealed that YM201636 treatment induced a striking increase in the intensity of perinuclear Rab7 (herein referring to Rab7a) immunoreactivity (Fig. 6A), resembling the perinuclear accumulation of LAMP1 in CA1 neurons of AD patients (Fig. 1A,B). These Rab7-immunopositive vesicles included enlarged vacuoles within the soma, which were decorated with Rab7 immunoreactivity (Fig. 6A, zoom), as is typical for PIKfyve inhibition in many cell types (Bissig et al., 2017; Chen et al., 2017; Choy et al., 2018; Dong et al., 2010; Ikonomov et al., 2002, 2001; Jefferies et al., 2008; Kim et al., 2014; Martin et al., 2013; McCartney et al., 2014) and is similar to vesicles seen in the CA3 region of AD patients (Fig. 1C). Specifically, YM201636 treatment exhibited a dose- and time-dependent effect to markedly and significantly increase the number and size of Rab7-immunopositive vesicles in primary neurons (Fig. 6A).

Notably, the colocalisation of Rab7 within and surrounding the enlarged vacuoles was the most selective when comparing it with that of several other endosomal-lysosomal markers including Rab5 (herein referring to Rab5a–Rab5c), EEA1 and lysobisphosphatidic acid (LBPA) (Fig. S3). Importantly, we further show that co-treatment of primary neurons with 4 µM YM201636 and 50 µM ML-SA1 led to both a significant reduction in the intensity of Rab7 immunopositive vesicles in the perinuclear region and a highly significant reduction in the number of vacuoles that were immunolabelled with Rab7 at 24 h, both of which were restored to control levels (Fig. 6B). Together our results indicate that reducing levels of the TRPML1 agonist PI(3,5)P_2_ induces an increase and enlargement of the endolysosomal compartments in neurons, similar to the endolysosomal pathology observed in LOAD hippocampal neurons (Fig. 1), and that this endolysosomal pathology can be remediated by ML-SA1 (Fig. 6B).

### Inhibition of PIKfyve replicates AD-like enlargement of early endosomes that is rescued by the TRPML1 agonist ML-SA1

**Fig. 7.**
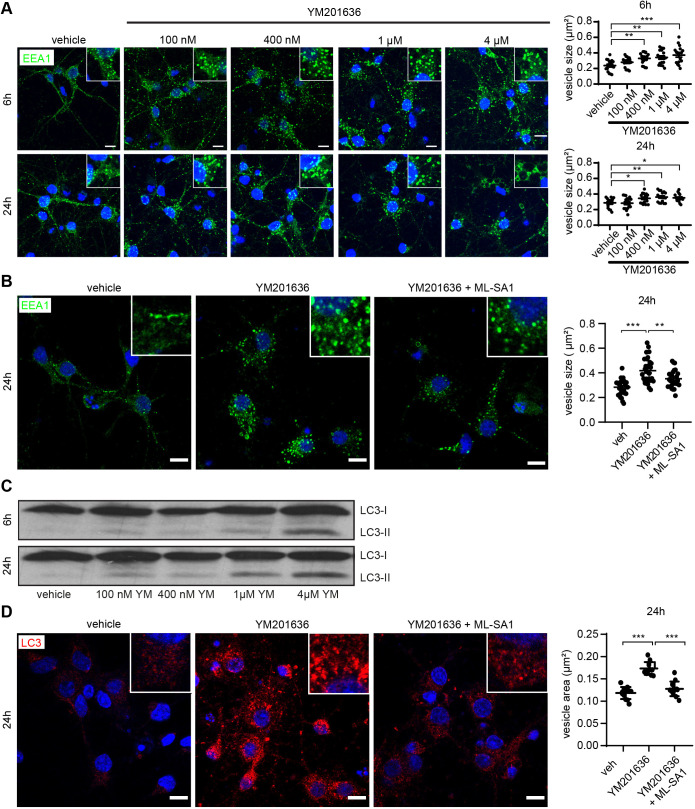
**Enlargement of EEA1-positive endosomes and increase in autophagy are dose dependent for YM201636 and can be rescued by ML-SA1 co-treatment.** (A,C) Representative images (left) and quantification (right) showing that depletion of PI(3,5)P_2_ using 100 nM–4 µM YM201636 (YM) in rat primary cortical neurons led to a dose-dependent increase in the size and intensity of early (EEA1) endosomes as well as to an accumulation of the autophagic marker LC3-II (C). Image in C representative of three repeats. (B,D) Representative confocal images (left) and quantification (right) showing early endosomal enlargement of EEA1-positive vesicles (B) and accumulation of LC3-II-positive autophagic vesicles (D) in rat primary cortical neurons after PIKfyve inhibition by 4 µM YM201636, which is restored by co-treatment with 50 µM ML-SA1. Scale bars: 10 µm. Quantitative data is based on four separate experiments with three images for each condition from two separate coverslips. *P<0.05; **P<0.01; ***P<0.001 (one-way ANOVA followed by Bonferroni post-hoc test). Data are expressed as mean±s.e.m.

Early endosomal swelling has been reported repeatedly as a very early event in AD neuronal pathology (Cataldo et al., 2000; Decourt et al., 2013; Nixon et al., 2001) using several early endosome markers including EEA1, Rab4 (Rab4a and Rab4b) and Rab5. Furthermore, pathological Rab5 activation has been shown to mimic AD-like endosomal dysfunction (Pensalfini et al., 2020). We investigated whether YM201636 would also induce enlargement of early endosomes in primary neurons, and whether this could be rescued by ML-SA1 co-treatment, by measuring EEA1 immunopositive vesicle size automatically using CellProfiler. Vehicle treated early endosomes were on average 0.28±0.013 µm^2^ (mean±s.e.m.). Incubation with YM201636 demonstrated a dose-dependent increase in EEA1 vesicle size to a maximum of 0.42±0.019 µm^2^ (Fig. 7A). Notably, co-treatment with 4 µM YM201636 and 50 µM ML-SA1 rescued this AD-like early endosome enlargement partially by reducing the size of early endosomes to 0.35±0.015 µm^2^ at 24 h (Fig. 7B).

### Inhibition of PIKfyve leads to a dose-dependent accumulation of autophagic vesicles that can be rescued by the TRPML1 agonist, ML-SA1

The presence of various types of electron-dense autophagic vesicles (AVs) has been reported by us and others in post-mortem neocortex and hippocampus of AD cases (Boland et al., 2008; Bordi et al., 2016; Tang et al., 2015). We investigated whether inhibition of PIKfyve increases autophagy in rat primary cortical neurons by measuring levels of LC3-II (the lipidated form of MAP1LC3 family proteins), a marker of autophagic vesicles. After 6 h, treatment with 1 and 4 µM YM201636, LC3-II levels increased slightly, whereas after 24 h a clear dose-dependent YM201636 (400 nM to 4 µM) increase in LC3-II was observed (Fig. 7C). This was supported by immunofluorescence data showing a strong increase in LC3 foci in primary neurons treated with 4 µM YM201636 for 24 h (Fig. 7D). In concordance with the ability of TRPML1 activation to rescue early endosomal and endolysosomal AD-like EAL pathologies, ML-SA1 abolished the accumulation of LC3-positive autophagic puncta in rat primary cortical neurons (Fig. 7D). This was confirmed measuring total protein levels by western blot analysis (data not shown).

## DISCUSSION

In this study, we demonstrate endolysosomal neuropathology in the brains of individuals who have had LOAD, indicative of functional defects in the endolysosomal TRPML1 Ca^2+^ channel. We further reveal a diminished ability of TRPML1 to release Ca^2+^, resulting in significant increases in endolysosomal Ca^2+­^ in iPSC-derived human neurons expressing APOE ε4*,* the greatest risk factor for LOAD. Our results show that blocking the biosynthesis of PI(3,5)P_2_, the endogenous agonist of TRPML1, in primary neurons by inhibiting PIKfyve, recreated TRPML1 endolysosomal neuropathology similar to that evident in LOAD neurons and APOE ε4 iPSC neurons. In addition, this treatment induced enlargement of early endosomes and the accumulation of autophagic vesicles, known to be central to EAL neuropathogenesis in AD. Finally, we demonstrate that the AD-like EAL neuropathology induced by PIKfyve inhibition can be remediated by treatment with ML-SA1, a small-molecule TRPML1 agonist. Together, these results highlight key defects in the TRPML1 endolysosomal system in AD pathogenesis and point to TRPML1 as a novel therapeutic target to remediate EAL neuropathogenesis in AD and related neurodegenerative disease.

TRPML1 is a master regulator of EAL health, whose malfunction causes neurodegeneration (Boudewyn and Walkley, 2019). Alterations in TRPML1 function have been associated with deletion of the PS-1 gene, implicating TRPML1 in FAD (Lee et al., 2015, 2010; Lie et al., 2022). However, little is known about TRPML1 function in LOAD. In our study, analysis of late endolysosomal health in LOAD hippocampal sections staged for AD severity implicates TRPML1 abnormalities in AD pathogenesis. We found levels of the endolysosomal marker LAMP1 are increased in CA1 and CA3 regions of AD hippocampi, and are enriched around senile plaques, consistent with previous analysis in the AD frontal cortex (Barrachina et al., 2006; Bordi et al., 2016; Piras et al., 2016). We demonstrate, for the first time to our knowledge, that there is a pronounced perinuclear clustering of endolysosomes in hippocampal AD neurons and, moreover, that a significant vacuolation of endolysosomal compartments is evident within CA3 cells with the phenotype of neurons in the AD brain. Increased perinuclear clustering of endolysosomes was also observed in GFAP-positive astrocytes in the AD hippocampus. Interestingly, studies indicate that TFEB function, a master regulator of lysosomal health, controlled by TRPML1, is defective in astrocytes in the AD brain (Bordi et al., 2016; Grubman et al., 2019; Martini-Stoica et al., 2018).

The perinuclear accumulation of endolysosomes in AD hippocampi, including their vacuolation, resembles changes in endolysosomal morphology that occur upon decreased activation of TRPML1 (Dong et al., 2010; Zhang et al., 2012). In addition, inhibition of PIKfyve kinase activity, including via loss of function of FIG4 and Vac14, key components of the PIKfyve complex, depletes cells of the vital TRPML1 agonist PI(3,5)P_2_. This causes a number of defects in the endolysosomal system that are similar to those in MLIV, the most prominent of which is vacuolated endolysosomal compartments (Bissig et al., 2017; Chen et al., 2017; Choy et al., 2018; Dong et al., 2010; Edgar et al., 2020; Ikonomov et al., 2002, 2001; Jefferies et al., 2008; Kim et al., 2014; Lines et al., 2017; Martin et al., 2013; McCartney et al., 2014; Zou et al., 2015).

Multiple risk genes for LOAD, including APOE ε4, play central roles in EAL function (Gao et al., 2018; Karch and Goate, 2015; Pimenova et al., 2018; Van Acker et al., 2019). Previous studies have shown that APOE ε4-encoding alleles cause EAL enlargement, and endolysosomal trafficking impairments *in vivo* (Nuriel et al., 2017; Xian et al., 2018), indicative of TRPML1 dysfunction, but whether APOE ε4, the greatest genetic risk factor for LOAD, promotes TRPML1 defects was completely unknown. We found that APOE ε4 leads to a diminished Ca^2+^ release via TRPML1 in isogenic human iPSC-derived cortical neurons, resulting in significant accumulation of endolysosomal Ca^2+^. These TRPML1-induced defects in endolysosomal Ca^2+^ handling were not accompanied by altered endolysosomal pH, indicating that endolysosomes in APOE ε4 neurons are not de-acidified. This was further indirectly verified by our finding that cathepsin D activity was increased in APOE ε4 neurons, in concordance with previous research showing increased cathepsin D levels in AD neurons (Cataldo et al., 1995, 1991). It is possible that endolysosomal levels of Fe^2+^ and Zn^2+^, or their release from endolysosomes, could be altered due to the TRPML1 defects we describe in APOE ε4 neurons. We did not investigate this is detail; however, our results show no significant difference in endolysosomal Zn^2+^ levels when comparing the cortical neurons with differing *APOE* isoform expression. Studies that interrogate TRPML1 regulation of endolysosomal Fe^2+^ homoeostasis in APOE ε4 cells and AD are an area deserving future investigation.

The pathological endolysosomal enlargement evident in AD neurons, which is also apparent in APOE ε4-expressing cells (Nuriel et al., 2017; Xian et al., 2018), coupled with the disruption of TRPML1-mediated Ca^2+^ efflux that we describe here for the first time, supports the idea that there is an inability to effectively regulate lysosomal fusion–fission cycles, which are essential for regulation of lysosome number, size and function, in AD (Bissig et al., 2017; Li et al., 2016; Saffi and Botelho, 2019). In addition, this would be predicted to impact other key endolysosomal and autophagic functions that are regulated by TRPML1, as this endolysosomal Ca^2+^ channel maintains the dynamic homeostasis of the EAL system (reviewed in Di Paola and Medina, 2019; Di Paola et al., 2018; Huang et al., 2020; Li et al., 2019; Venkatachalam et al., 2015; Waller-Evans and Lloyd-Evans, 2015). Furthermore, recent studies show TRPML1 regulates broader cell functions, especially inter-organellar Ca^2+^ signalling (Kilpatrick et al., 2013). This includes regulation of mitochondrial Ca^2+^ dynamics (Calvo-Rodriguez et al., 2020; Jadiya et al., 2019) and ryanodine-receptor 2 (RyR2) Ca^2+^ release function (Thakore et al., 2020), which are known to be impaired in AD neurons (Chami and Checler, 2020; Kelliher et al., 1999; Lacampagne et al., 2017).

We were unable to obtain clear endolysosomal immunoreactivity with multiple LAMP1 antibodies in primary rat neurons and thus we employed the endolysosomal marker Rab7. LAMP1 is abundant on the lysosomal membrane and is most commonly used to label lysosomes (Wartosch et al., 2015). Rab7 is mechanistically important in membrane transport from the late endosome to the lysosome and is reported to label endolysosomal compartments where it shows a strong colocalization with LAMP1 (Bucci et al., 2000; Guerra and Bucci, 2016; Humphries et al., 2011). Although LAMP1 and Rab7 colocalize in several endolysosomal compartments they can also label mutually exclusive membrane compartments, and the endolysosomal compartment in neurons has an added complexity (Lie et al., 2021). These considerations should be acknowledged when comparing our findings on endolysosomal integrity in the human brain and rat cortical neurons.

Here, we tested the hypothesis that depleting rat primary cortical neurons of PI(3,5)P_2_, the TRPML1 agonist, by inhibiting PIKfyve kinase activity using the pharmacological inhibitor YM201636, would recreate the key EAL defects we detected in AD brain and in human neuronal APOE ε4 iPSCs. We found that PIKfyve inhibition increased endolysosomal Ca^2+^ levels to those we found in APOE ε4*-*expressing neurons and was associated with the marked vacuolation of endolysosomal compartments. We further demonstrated that blocking PIKfyve activity induced a significant increase in the size of early endosomes in neurons. Notably, early endosomal swelling is a very early event in AD neuropathology (Cataldo et al., 2000; Decourt et al., 2013; Nixon et al., 2001). Finally, inhibiting neuronal PIKfyve activity significantly increased the number of LC3-positive autophagic puncta, which is in concordance with the presence of various types of electron-dense autophagic vesicles (AVs) reported in post-mortem neocortex and hippocampus of AD cases (Boland et al., 2008; Bordi et al., 2016; Tang et al., 2015).

Induction of these broad AD-like EAL defects in neurons by PIKfyve kinase inhibition has not been reported previously. However, recent studies found that loss of PIKfyve due to prion infection drives the spongiform neurodegeneration and neuronal vacuolation in prion disease, which can be rescued by PI(3,5)P_2_ supplementation (Lakkaraju et al., 2021). Other recent work demonstrates that pharmacological inhibition of PIKfyve using YM201636 and apilimod reduces the trafficking of tau and α-synuclein from early endosomes to late endolysosomes, thus preventing fibril formation and implicating PIKfyve inhibition as neuroprotective in these *in vitro* models (See et al., 2021 preprint; Soares et al., 2021). However, the degree of EAL defects we demonstrate in neurons upon PIKfyve inhibition would advise caution in using PIKfyve inhibitors to protect against tau or α-synuclein fibrillization and spread in neurodegenerative disease, as also discussed by Lakkaraju et al. (2021). Collectively, these studies draw attention to the potential role of the PIKfyve complex in EAL defects in AD and other related neurodegenerative diseases. With respect to AD, it has been shown that the amyloid precursor protein (APP) binds to the PIKfyve complex and can regulate PIKfyve function and the formation of PI(3,5)P_2_ (Balklava et al., 2015; Currinn et al., 2016). Endogenous levels of the low abundance PI(3,5)P_2_, which localizes to the late endolysosome, cannot be detected with current mass spectrometry technology. However, our results show alterations in PI dynamics in AD brain, selectively affecting two PI species with broad functions in EAL trafficking and function, namely PI(3,4,5)P_3_ and PI(4,5)P_2_ (Balla, 2013; Botelho, 2009; Di Paolo and De Camilli, 2006; Vanhaesebroeck et al., 2012), the latter of which operates as the endogenous antagonist of TRPML1. Importantly, several LOAD risk genes were shown to regulate enzymes that control PI dynamics and interconversion (reviewed in Raghu et al., 2019), and altered PI composition has been reported previously in the LOAD brain (Morel et al., 2013; Stokes and Hawthorne, 1987; Zhu et al., 2015). Very recent research suggests that it is possible to achieve independent measurement of PIP_2_ regioisomers, enabling measurement of PI(3,5)P_2_ (Morioka et al., 2022). Achieving this should enable further understanding of mechanisms by which the dynamics of this vital low abundance phosphoinositide couples late endolysosomal trafficking and function to TRPML1 activation in health and neurodegenerative disease.

Remarkably, we demonstrated for the first time that TRPML1 activation, via the small-molecule agonist ML-SA1 (Fine et al., 2020; Grimm et al., 2010; Shen et al., 2012), overrides the PI(3,5)P_2_ deficit-induced defects caused by PIKfyve inhibition in neurons and protects against multiple AD-related EAL neuropathologies. In agreement, TRPML1-induced endolysosomal Ca^2+^ release ameliorated some endolysosomal defects when PIKfyve activity was inhibited in other cell types (Edgar et al., 2020; Li et al., 2016; Lines et al., 2017; Zou et al., 2015). Interestingly, here we demonstrated a much broader impact of ML-SA1 to remediate defects in EAL machinery induced by PIKfyve inhibition in primary neurons. Thus, ML-SA1 enabled normal Ca^2+^ release from endolysosomes and restored the size of lysosomal Ca^2+^ stores, diminished late (Rab7) and early (EEA1) endosomal enlargement and reduced the number of LC3-positive autophagic puncta. Taken together, these results highlight the mechanistic link between PIKfyve-PI(3,5)P_2_ and TRPML1 in maintaining EAL neuronal health.

Interestingly, a protective effect of ML-SA1 has been demonstrated against α-synuclein toxicity in human dopaminergic neurons (Tsunemi et al., 2019), L-BMAA-induced neurodegeneration, modelling ALS, in primary neurons and in FIG4 deficiency linked to Charcot–Marie–Tooth disease (Edgar et al., 2020; Zou et al., 2015). These results suggest that ML-SA1 remediation of FIG4 deficiency and PIKfyve inhibition are mechanistically linked to activation of TRPML1-induced lysosomal fission. Furthermore, ML-SA1 cleared sphingomyelin and Aβ from LAMP1-positive lysosomes in an HIV cell model (Bae et al., 2014), and ML-SA1-induced acidification of endolysosomes blocked the LDL-induced increase in intra-neuronal and secreted levels of Aβ (Hui et al., 2019). Hui et al. further showed that antiretroviral drugs increase Aβ levels by de-acidifying endolysosomes, and that ML-SA1 prevented the resulting Aβ accumulation (Hui et al., 2021). Furthermore, TRPML1 activation is essential for TFEB-mediated regulation of lysosomal exocytosis, reducing tau pathology and spread in animal models (Xu et al., 2020).

Together, our study reveals that the TRPML1 agonist ML-SA1 reverses multiple key EAL abnormalities in primary neurons caused by PIKfyve inhibition that are similar to those described in AD neurons. These findings provide clear implications for our improved understanding of abnormalities in the EAL system in AD and other neurodegenerative diseases, highlight the mechanistic importance of TRPML1 endolysosomal Ca^2+^ signalling in these diseases and identify TRPML1 as a target for therapeutic intervention.

## MATERIALS AND METHODS

### Antibodies and reagents

The following antibodies were used for immunofluorescence: anti-EEA1 (BD Biosciences, USA, #610456, 1:400), anti-LAMP1 (D2D11) (Cell Signaling, USA, #9091, 1:50), anti-LAMP1 (H4A3) (Abcam, UK, ab25630 1:50); anti-LC3 (Cell Signaling, #2775, 1:250), anti-p-Ser396/404 tau (PHF1, a generous gift from Dr Peter Davies, Albert Einstein College of Medicine, NY, USA, 1:200), anti-Rab7 (Santa Cruz Biotechnology, USA, sc-376362, 1:100), anti-GFAP (DAKO, Z0334, 1:200), anti-TRPML1 [Sigma, HPA031763, batch 2 (2017) 1:10], goat-anti-mouse-IgG conjugated to Alexa Fluor 488 (Thermo Fisher Scientific, USA, A11001, 1:400), donkey-anti-rabbit-IgG conjugated to Cy3 (Jackson Laboratories, USA, #711-165-152, 1:400). The following antibodies were used for western immunoblot analysis: anti-LAMP1 (H4A3) (Abcam, ab25630, 1:5000), anti-LC3 antibody (Cell Signaling, US, #2775, 1:1000), anti-TRPML1 [Sigma, HPA031763, batch 1 (2016) 1:250, batch 2 (2017) 1:1000], horse-anti-mouse-IgG conjugated to HRP (Cell Signaling, #7076, 1:1000) and goat anti-rabbit-IgG conjugated to HRP (Cell Signaling, #7074, 1:1000). The following reagents were used: B27™ Supplement (Invitrogen), BODIPY-Pepstatin (Invitrogen, P12271), DNase1 (Sigma-Aldrich), FITC–dextran (Sigma-Aldrich, FD10S), Fluozin-3-AM (Invitrogen, F24195), Fura2-AM (Invitrogen), GlutaMAX™ (Invitrogen), Hank's balanced salt solution (HBSS) (Invitrogen), HEPES (Gibco, USA), ML-SA1 (Sigma-Aldrich, #SML0627), GW405833 (Sigma-Aldrich, #G1421), Neurobasal medium (Invitrogen, USA), Oregon Green™ 488 BAPTA-1, AM (Invitrogen, O6812), Pluronic™ F-127 (Sigma Aldrich, US, P2443), Poly-_D_-lysine (Sigma Aldrich, US), papain (Worthington, US), Pen/Strep (Invitrogen, US), sodium pyruvate (Invitrogen, US), Texas Red-dextran (Invitrogen, US, D1863), YM201636 (Invivogen, US, #INH-YM20).

### Brain tissue

Brain tissue was provided by the Netherlands Brain Bank (NBB; see Table 1 for case details). Ethical approval and written informed consent from the donors or the next of kin was obtained in all cases (Griffin et al., 2005; Moloney et al., 2010) and all clinical investigation have been conducted according to the principles expressed in the Declaration of Helsinki. The work of the NBB abides by the ethical code of conduct approved by the Ethics committee and strict ethical guidelines as stated in Brain Net Europe Ethical Code of Conduct for brain banking (Klioueva et al., 2015). Clinical diagnosis of probable Alzheimer's disease was made according to the NINCDS–ADRDA criteria, and severity of dementia rated by the Global Deterioration Scale. Non-demented controls had no history or symptoms of neurological or psychiatric disorders. All cases were neuropathologically confirmed, using conventional histopathological techniques, and diagnosis performed using the CERAD criteria. Neuropathological staging of neurofibrillary changes (0–VI) was performed according to Braak and Braak (Braak and Braak, 1991). The degree of Aβ deposition in neuritic senile plaques was assessed in the temporal cortex, indicated as 0, A, B and C, for no, mild, moderate and high levels of senile plaques, respectively. Tissue fractions for western immunoblot and phosphoinositide analysis were prepared from AD and matched control mid-temporal cortex samples as described below. Brain tissue for immunofluorescence analysis was provided as formalin fixed and paraffin-embedded 8-µm-thick consecutive sections prepared on Superfrost slides as previously described (Moloney et al., 2010). Control and AD tissue was matched for post-mortem delay, tissue pH, age, and agonal status as described previously (Griffin et al., 2005; Moloney et al., 2010). Western blot analysis using anti-TRPML1 batch 1 (2016) antibody was performed on brain tissue published previously in (Moloney et al., 2010) as well as tissue listed in Table 1.

### Preparation of tissue fractions

Brain material for western immunoblotting was obtained as ∼1 g frozen pieces, which were thawed, homogenised and fractionated. Tissue fractions were prepared as previously described (Griffin et al., 2005; Moloney et al., 2010). Briefly, membrane-enriched fractions (100,000 ***g*** pellet) were separated from the soluble cytosolic fractions (100,000 ***g*** supernatant) following centrifugation of tissue homogenates in a Beckman ultracentrifuge (type 42.1 rotor) at 100,000 ***g*** for 60 min at 4°C. Tissue fractions were stored at −70°C.

### Human iPSC culture and neural differentiation

The isogenic APOE ε2/ε2 (BIONi010-C-6), APOE ε3/ε3 (BIONi010-C-2), APOE ε4/ε4 (BIONi010-C-4) and *APOE*-null (BIONi010-C-3) iPSC lines were obtained from the EBiSC stem cell repository (https://cells.ebisc.org). iPSCs were cultured on vitronectin (Life Technologies)-coated six-well plates with E8 flex medium (Life Technologies) at 37°C and 5% CO_2_. The medium was changed 1 day after plating and subsequently every other day until cells were 60–70% confluent. On the day of embryoid body (EB) formation, cells were washed once with PBS, treated with ReLeSR (Stem Cell Technologies, USA), collected as clumps, and transferred to a non-adherent dish where they were maintained overnight. The next day, EBs were washed with PBS and cultured on SLI medium, which contained advanced DMEM F-12 medium (ADF) supplemented with GlutaMAX™, penicillin and streptomycin (Life Technologies), 2% NeuroBrew 21 without retinoic acid (Miltenyi Biotec, Germany), the SMAD pathway inhibitors LDN193189 (1μM, Stemgent, USA) and SB431542 (10μM, Abcam), and the WNT pathway inhibitor IWR1 (1.5μM) (Tocris, UK; Chambers et al., 2009). On day 6, the medium was replaced by SB431542- and LDN193189-containing medium without IWR1. On day 8, cells were treated with ReLeSR and plated onto a Matrigel (Corning, USA)-coated dish and cultured to day 16 in NMM medium [ADF with 2% Neuro Brew 21 with retinoic acid (Miltenyi Biotec) and 10 ng/ml FGF]. Neural progenitor cells were either frozen or further expanded.

Neuronal differentiation was initiated by seeding neuronal progenitor cells on a substrate of growth factor-reduced Matrigel and poly-L-lysine (Sigma-Aldrich) at a density of 10^5^ cells/cm^2^ and cultured for 7 days in SynaptoJuice A medium which contained ADF, 2% NeuroBrew 21 with retinoic acid, 2 µM PD0332991 (Selleckchem, USA), 10 µM DAPT (Sigma-Aldrich), 10 ng/ml BDNF (Miltenyi Biotec), 500 nM LM22A4 (Tocris), 10 µM Forskolin (Tocris), 3 µM CHIR99021 (Tocris), 300 µM GABA (Sigma-Aldrich), 1.8 mM CaCl_2_ (Sigma-Aldrich) and 200 mM ascorbic acid (Sigma-Aldrich). Half of the medium was refreshed every 2–3 days. After 7 days, the medium was replaced by SynaptoJuice B medium, which contained ADF and Neurobasal A medium in equal volume, 2% NeuroBrew 21 with retinoic acid, 2 µM PD0332991, 10 ng/ml BDNF, 1.8 mM CaCl_2_ and 200 mM ascorbic acid. Neurons were kept in SynaptoJuice B for up to 2 weeks, refreshing half the medium every 2–3 days. Extensive characterization of neurons obtained using this protocol has been published previously in various iPSC lines (Telezhkin et al., 2016). Cells were routinely monitored for mycoplasma and bacterial or yeast contaminations.

### Primary cortical neuron culture

Primary cortical neurons were derived from embryonic day 18 wild-type Sprague-Dawley rat embryos as previously described (Boland et al., 2008). All animal experiments were performed according to approved guidelines. Single-cell suspensions obtained from cortices of individual embryos were plated on a poly-D-lysine-coated surface in neurobasal medium supplemented with 0.5 mM GlutaMAX™, 50 U/ml penicillin-streptomycin and 2% B27™ Supplement (50×). Cortical neurons were maintained at 37°C and 5% CO_2_, with half of the plating medium being replaced every 3 days with neurobasal medium supplemented with 0.5 mM GlutaMAX™ and 2% B27 supplement (50×). Cortical neurons were cultured for 6 to 8 days *in vitro* (DIV6–8) before drug treatments were applied. Neurons were treated with YM201636 (Invivogen) or ML-SA1 (Sigma-Aldrich) diluted in neuronal medium for the time and concentration indicated in the results section. Cells were routinely monitored for bacterial or yeast contaminations.

### Ca^2+^ measurements

Neuronal iPSCs were plated in 8-well chamber slides (Ibidi, Germany) and loaded with 1 µM Fura-2-AM in culture medium containing 1% BSA at room temperature for 30–60 min, then washed with 1× HBSS with 10 mM HEPES, 1 mM MgCl_2_ and 1 mM CaCl_2_, left for 10 min to allow de-esterification of the Ca^2+^ dye, and imaged in 1× HBSS with 10 mM HEPES, 1 mM MgCl_2_ and 50 µM CaCl_2_. Fluorescence was recorded using a Zeiss Axiovert 35 microscope with a CAIRN Optospin filter wheel, EXFO X-cite 120Pc light source and an ORCA-flash4.0 LT camera at two different excitation wavelengths (340 and 380 nm) and a single (495 nm) emission wavelength. Videos were recorded using the MetaFluor software (Molecular Device, Sunnyvale, CA, USA) and the ratio (*F*_340_/*F*_380_) was used to detect changes in intracellular [Ca^2+^] from whole-cell ROIs with background subtracted. Changes in cytoplasmic Ca^2+^ levels were recorded after addition of 2 µM ionomycin (Calbiochem, USA) to clamp non-lysosomal stores followed by addition of 500 µM GPN (AlfaAesar, USA) to release lysosomal Ca^2+^ or 200 nM bafilomycin A1 (Sigma-Aldrich) to activate TRPML1 via mild deacidification. Cells were pre-treated for 10 min with 10 µM GW405833 (Sigma-Aldrich) for TRPML1 inhibition and 5 µM xestospongin-C (Sigma Aldrich) for IP_3_ receptor inhibition where needed. Cells were not pre-treated with ionomycin or xestospongin to record Ca^2+^ release via 10 µM ML-SA1 alone, to capture the full physiological cellular response including subsequent Ca^2+^ release from other compartments. Ca^2+^ measurements on primary rat cortical neurons were carried out in a similar fashion but using 5 µM Fura-2-AM and an Olympus IX51 inverted fluorescence microscope equipped with a 75-W xenon arc lamp, an Optiscan monochromator (Cairn, Kent, UK), an Orca-ER charged-coupled device (CCD) camera (Hamamatsu Photonics, Hertfordshire, UK), and an Olympus UplanF1 1.3 NA 100× oil-immersion objective. Images were acquired and analysed with Andor iQ Bioimaging software version 1.9 (Andor, Belfast, UK) including subtracting of background by masking. *In situ* Ca^2+^ levels were measured as previously described (Lloyd-Evans et al., 2008), but using the cell-impermeant high molecular mass Ca^2+^-sensitive OGB (0.5 mg/ml) alongside Texas Red–dextran (0.1 mg/ml, not sensitive to Ca^2+^) as loading control (protocol adapted from Lelouvier and Puertollano, 2011 to measure lysosomal Ca^2+^). The ratio of green (OGB) to red (Texas Red–dextran) fluorescence, when calibrated against luminal pH, is indicative of the luminal Ca^2+^ concentration in endolysosomes.

### Lysosomal pH assay

Neurons were loaded with 500 µg/ml pH-sensitive FITC–dextran (10,000 kDa) and 250 µg/ml Texas Red-dextran (10,000 kDa, non-pH-sensitive loading control) in 1:1 Advanced DMEM/F12 (with GlutaMax™): Neurobasal A (Life Technologies), 1% penicillin/streptomycin (Life Technologies), 2% NeuroBrew21 with Retinoic Acid (Miltenyi Biotec), 0.3 mM CaCl_2_ (to give 1.8 mM CaCl_2_ in final complete medium; Sigma-Aldrich) and 200 µM ascorbic acid (Sigma-Aldrich) for 24 h. Then dextran medium was removed, and cells were washed in fresh medium and incubated for a further 24 h in fresh medium. The medium was removed, and nuclei stained with Hoechst 33342 (1 µg/ml in warmed HBSS; Sigma-Aldrich) for 10 min, washed in HBSS and imaged using a Perkin Elmer Operetta high content imaging system. To determine the ratio of FITC/Texas Red fluorescence, cells were first identified using the Hoechst 33342 staining (352 nm/454 nm). Texas Red-positive vesicles within the cell body were identified, and fluorescence intensity for both Texas Red (586 nm/603 nm) and FITC (491 nm/516 nm) was measured within each vesicle. The FITC/Texas Red ratio was calculated for each vesicle and averaged per well. Three plates with two wells per plate were imaged for each *APOE* genotype.

### Subcellular distribution of cathepsin D

The subcellular distribution of processed active cathepsin D was analysed using BODIPY-pepstatin (Invitrogen) following the manufacturer's instructions, but at a 1:500 dilution and incubating for 15 min at 37˚C followed by three washes in Dulbecco's PBS (DPBS). Images were taken and analysed using a Perkin Elmer Operetta high content imaging system.

### Zn^2+^ measurement

Cells were stained with 5 µM Fluozin-3-AM (Invitrogen) [in SynaptoJuice B containing 0.0025% pluronic acid F127 (Sigma-Aldrich) dissolved in DMSO] for 30 min at 37˚C. Cells were washed twice and imaged in HBSS containing 1 mM CaCl_2_, 1 mM MgCl_2_ and 5 mM HEPES (pH 7.2). Images were taken on the Colibri LED microscope system at 470 nm (excitation)/510 nm (emission) or Perkin Elmer Operetta high-content imaging system.

### Western immunoblot analysis

A BCA protein assay (Millipore) was used for protein measurements. Cell and tissue extracts were run on 8–16% Tris-glycine gels in Tris-glycine running buffer and transferred onto 0.2 µm nitrocellulose membranes. LiCor total protein stain (LI-COR, USA) was used to ensure equality of protein loading and is presented in Fig. S4. After blocking in 5% non-fat milk or 5% BSA, membranes were processed for immunoblotting. Immunodetection was obtained using ECL reagent (GE Healthcare, USA) and developed using Fujifilm X-ray film.

### Immunofluorescence of brain sections

Immunofluorescence analysis was performed as described previously (Moloney et al., 2010). Briefly, sections were deparaffinised and hydrated prior to microwave pre-treatment (30 min in 10 mM citrate buffer pH 6.4). After cooling, nonspecific binding was blocked using 5% normal donkey serum (Sigma-Aldrich) in PBS pH 7.4 for 30 min at room temperature, followed by overnight incubation with primary antibodies in blocking buffer. Slides were then incubated in secondary antibodies diluted in blocking buffer for 1 h at room temperature in the dark. Nuclei were stained using 4′,6-diamidino-2-phenylindole (DAPI; 1.25 µg/ml,) (Sigma-Aldrich). To lower the intensity of lipofuscin auto-fluorescence, slides were finally incubated for 10 min with 0.1% Sudan Black B (Sigma-Aldrich) in 70% ethanol, washed with 1× PBS and mounted using fluorescence mounting medium (DAKO, USA). Images were visualised on a Leica DMI 3000B inverted fluorescence microscope and captured using a Leica DFC420C digital camera, and on an Olympus FluoView FV10i. Confocal images were captured on an inverted Zeiss LSM 510 Meta confocal microscope, using a PlanApo 63×1.6 N/A oil immersion objective. Images were processed using ImageJ/Imaris 7.2 (Bitplane, CH).

### Immunofluorescence of primary neuronal cultures

Cells were fixed (4% paraformaldehyde, 15 min, RT or MeOH, 10 min, −20°C), quenched (50 mM NH_4_Cl, 15 min, room temperature) and permeabilized (0.3% Triton-X-100 in 1% BSA-PBS, 10 min, room temperature) for indirect immunofluorescence. Incubation with primary antibodies in blocking solution (5% BSA-PBS, 2 h, room temperature) was followed by a 1 h incubation with Alexa Fluor-488- or Cy3-conjugated secondary antibodies (in 5% BSA-PBS, room temperature) and mounting in Mowiol. Images were captured using a Zeiss LSM 510 Meta confocal microscope system and oil-immersion Plan-Apochromat 63× A/1.40 NA objective lenses. Data were collected using Zeiss ZEN software and processed in ImageJ. Quantitative Rab7, EEA1 and LC3 data is based on four separate experiments with three images for each condition from two separate coverslips (24 images per treatment).

### Mass spectrometry analysis of phosphoinositide levels

Mass spectrometry was used to measure phosphoinositide lipid levels essentially as previously described (Furlong et al., 2019; Kielkowska et al., 2014), using a QTRAP 4000 (AB Sciex) mass spectrometer and employing the lipid extraction and derivatization method described for whole tissue (temporal cortex 0.5 mg wet weight ground tissue), with the modification that 10 ng C17:0/C16:0 PI(3,4,5)P_3_ internal standard (ISD) and 10 ng C17:0/C16:0 PI ISD were added to primary extracts. Measurements were conducted for an *n*=12 for human temporal cortex samples of AD patients and controls (Table 1). PIP, PIP_2_ and PIP_3_ response ratios were calculated by dividing the total PIP, PIP_2_ and PIP_3_ response areas for the most abundant molecular species present [C38:4+C38:3] in the cortex by the corresponding response areas of the PIP_2_−ISD (for PIP and PIP_2_) and PIP_3_−ISD (PIP_3_ only) in each sample. PI response ratios were calculated by dividing PI response areas by the response area for the PI−ISD. PIP, PIP_2_, and PIP_3_ response ratios were then normalised to the PI response ratio to account for any cell input variability. In some experiments, C38:4-PI(3,4)P_2_ and C38:4-PI(4,5)P_2_ regioisomers were distinguished and quantified in parallel ground cortex samples (0.5 mg wet weight), employing previously described methods (Malek et al., 2017).

### Statistical analysis

Data are expressed as means±s.e.m. and statistics are based on at least *n*=3 (biological replicates) if not stated otherwise. Significance levels for comparisons between two groups were determined using an unpaired two-tailed Student's *t*-test. Significance levels for comparisons between more than two groups were determined using one-way ANOVA, followed by the Bonferroni post-hoc test. A *P*-value of 0.05 was considered as the borderline for statistical significance. GraphPad Prism™ 8 and Microsoft Excel software were used for statistical analysis and generation of graphs. Significance is denoted as *P<0.05; **P<0.01; ***P<0.001.

## Supplementary Material

10.1242/joces.259875_sup1 Supplementary information
