# Supplementary material for: The synthetic TRPML1 agonist ML-SA1 rescues Alzheimer-related alterations of the endosomal-autophagic-lysosomal system
Source: J Cell Sci. 2023 Mar 21;136(6):jcs259875. doi: 10.1242/jcs.259875 (PMC10112969; doi:10.1242/jcs.259875)
Supplement: Supplementary information [file joces-136-259875-s1.pdf]

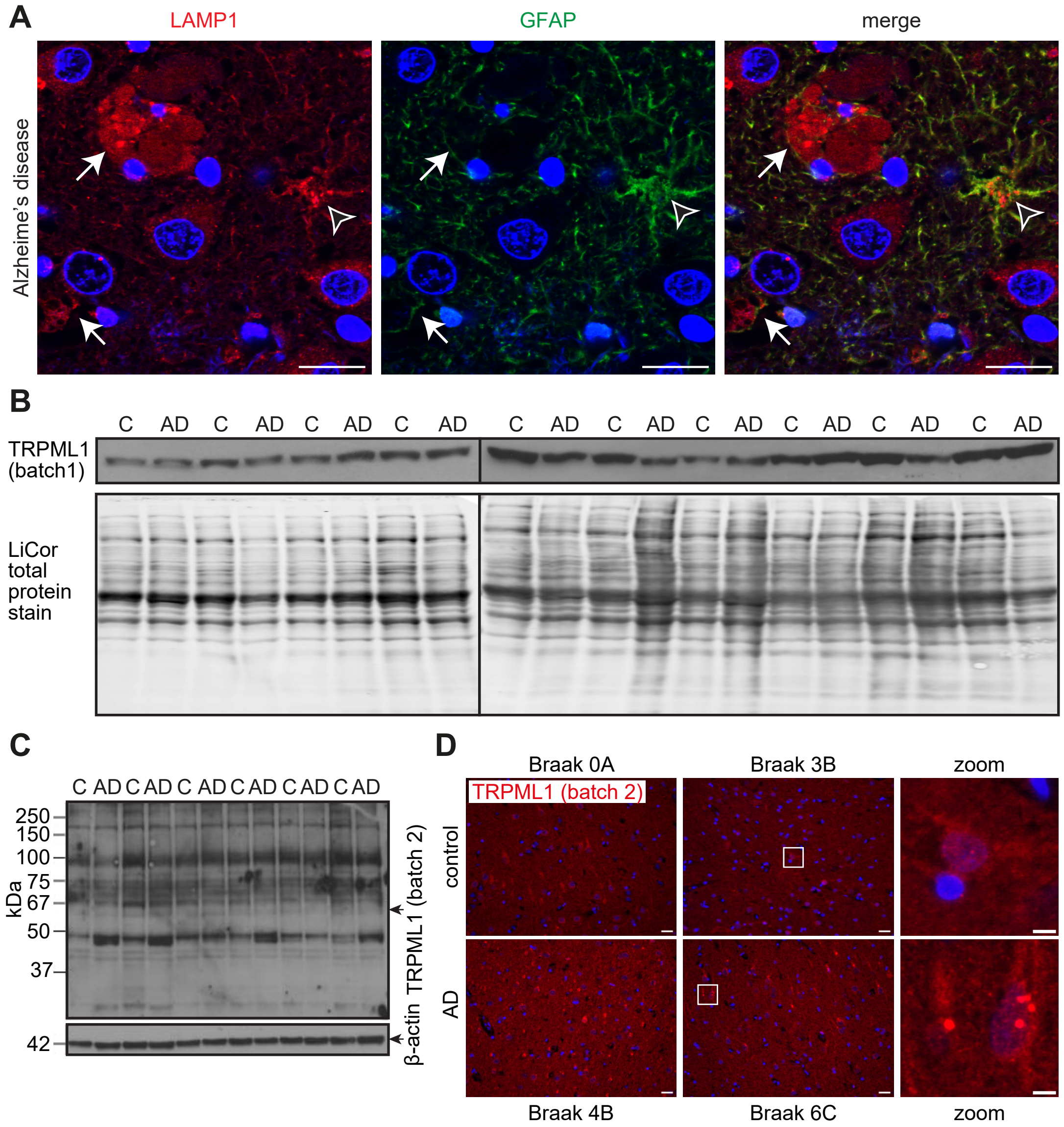

Fig. S1. Enlarged LAMP-1 and TRPML1-positive endolysosomes are found in AD brain tissue. (A) Representative images showing the accumulation and swelling of LAMP1-positive vesicles in large pyramidal neurons (arrows) and GFAP-positive glia cells (empty arrowhead) in the CA1 region of hippocampal sections of AD (n = 1). Scale bar 20 µm. (B) Western immunoblot analysis of temporal cortex membrane fractions (n = 10 from (Moloney et al., 2010) shows no difference in TRPML1 levels between AD patients and controls using batch 1 (2016) of anti-TRPML1 antibody HPA031763. (C) Western immunoblot analysis of temporal cortex membrane fractions (n = 6) using batch 2 (2017) of anti-TRPML1 antibody HPA03163 shows no clear TRPML1 band. LiCor total protein stain (B) or β-actin antibody staining (C) were used to ensure equal loading. (D) Representative images (left, middle) showing increased TRPML1-positive vesicles in large pyramidal neurons (zoom, right) in hippocampal sections of AD (n = 10), which are absent from control sections (n=7) using batch 2 (2017) anti-TRPML1 antibody HPA03163. Scale bar 20 µm (images) and 5 µm (zoom).

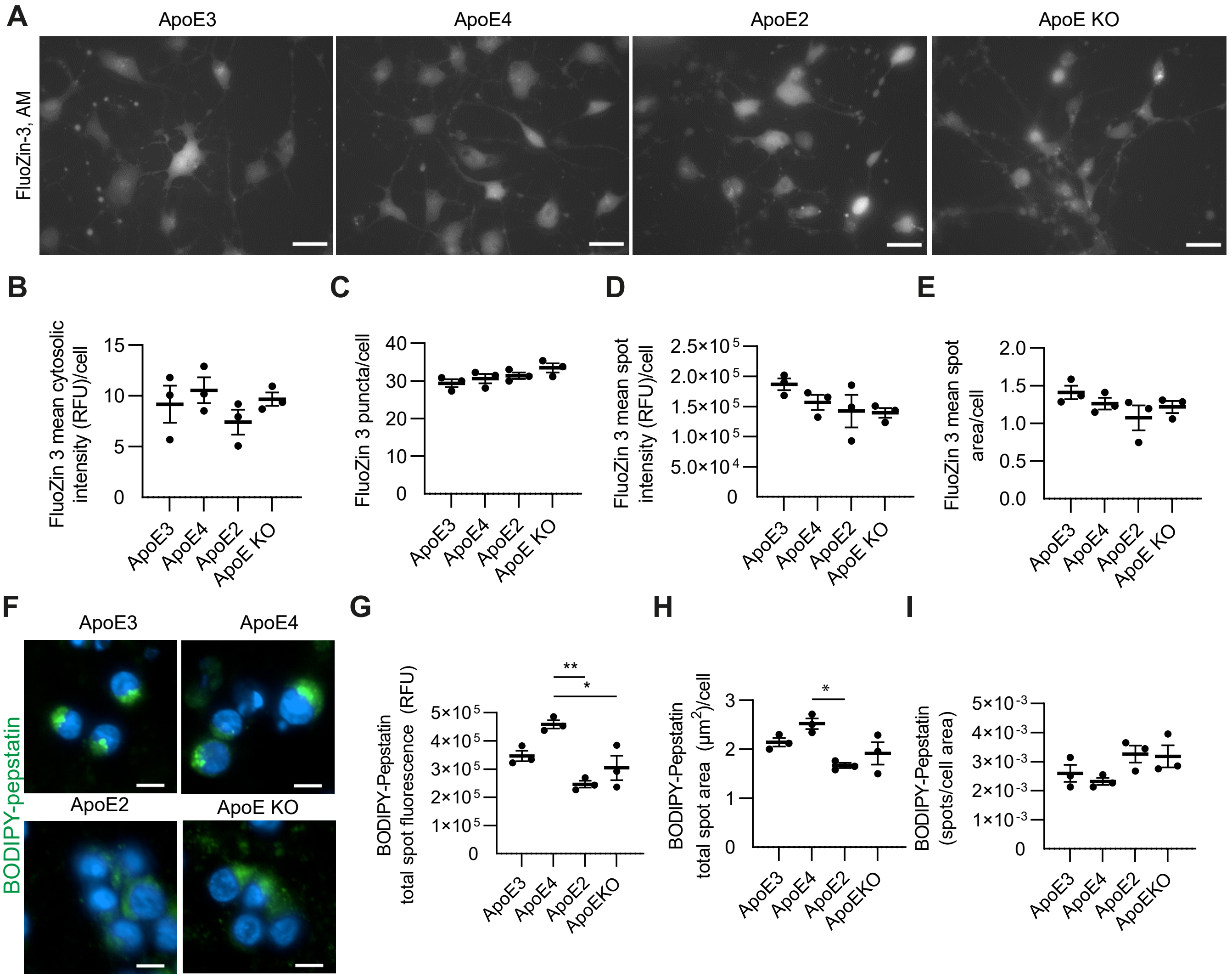

Fig. S2. Unaltered Zn^{2+} levels and increased cathepsin D activity in a neuronal LOAD iPSC model. (A-E) Representative images (A) and quantification (B-E) of *APOE* ε3, *APOE* ε4, *APOE* ε2 or *APOE*$^{-/-}$ iPS neurons loaded with FluoZin-3, AM showed no difference in mean cytosolic FluoZin3 intensity (B), FluoZin3 puncta-cell (C), FluoZin3 mean spot intensity (D) and FluoZin3 mean spot area/cell (E). Scale bar 20 μm. (F-I) Representative images (F) and quantification (G-I) of cathepsin D activity using BODIPY-Pepstatin showed an increase in total BODIPY-pepstatin spot intensity (G) and area/cell (H), but no change in the number of BODIPY-pepstatin spots/cell area (I) in *APOE* ε4 iPSC neurons. Scale bar 10 μm. Quantitative data is based on three biological replicates with one technical repeat. *, $p < 0.05$; **, $p < 0.01$; ***, $p < 0.001$. Data are expressed as means ± SEM values. Significance levels were determined with one-way ANOVA, followed by post-hoc Bonferroni test.

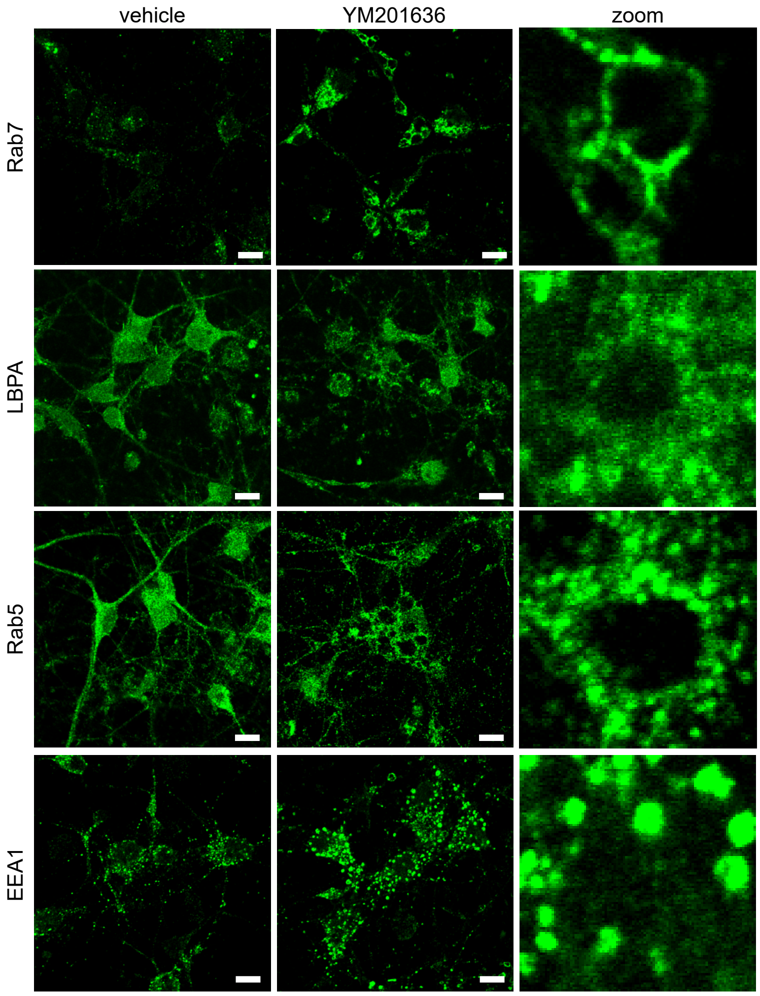

Fig. S3. YM201636-enlarged primary neuron vacuoles are decorated with Rab7, but not Rab5, EEA1 and LBPA (lysobisphosphatidic acid). Representative images of primary neurons treated with vehicle (left) or 4 µM YM201636 for 24h (middle and zoom right) and immunolabelled with Rab7 (n=4, 3 technical replicates), LBPA (n=5, 2 technical replicates), Rab5 (n=1, 2 technical replicates) or EEA1 (n=4, 3 technical replicates). Scale bar 20 µm.

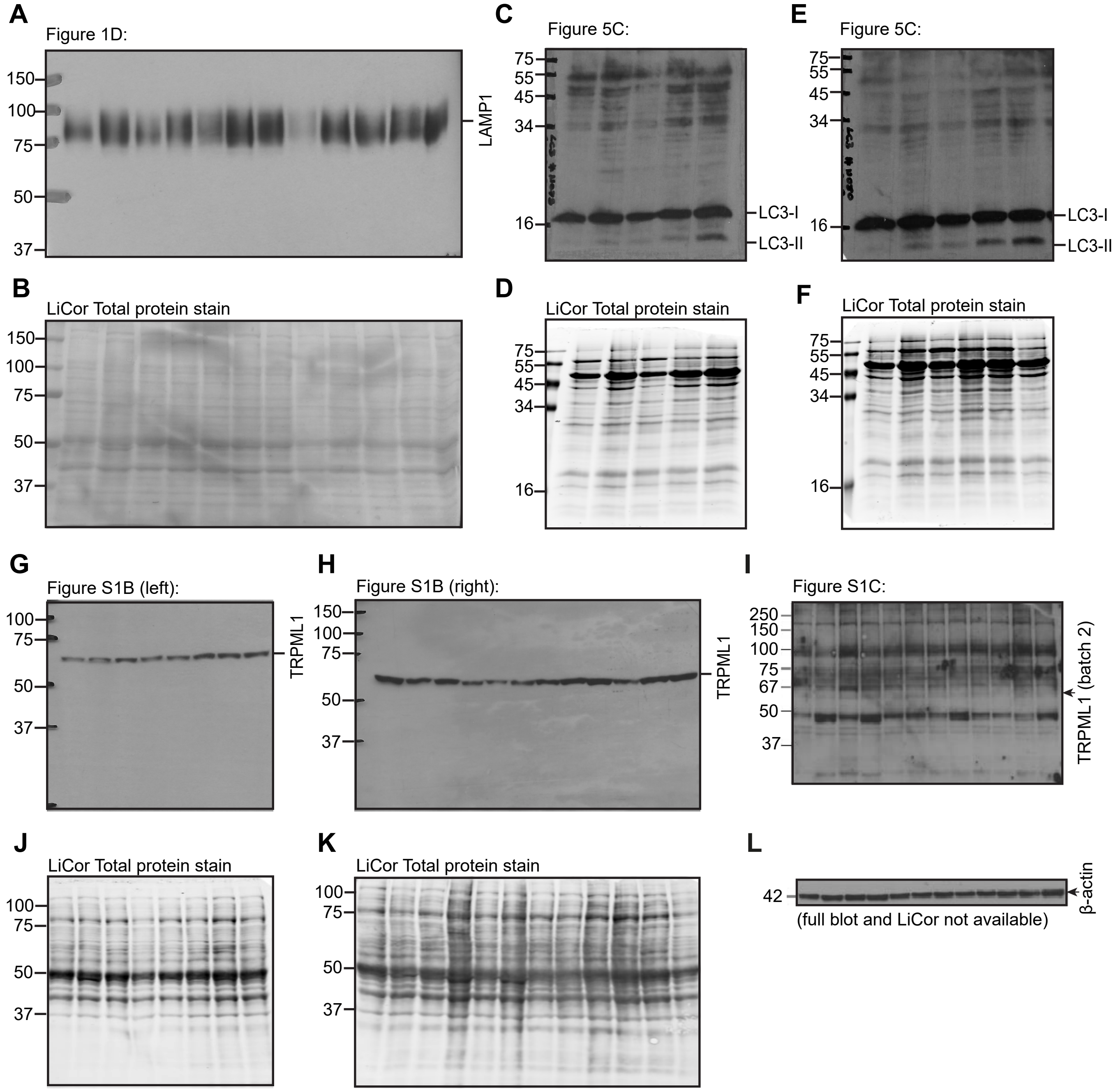

Fig. S4. Blot Transparency. Uncropped Western immunoblots of LAMP1 from Fig 1D (A), LC3 6h (C) and 24h (E) from Fig 5C, TRPML1 from Fig S1B (G, H, batch 1) and Fig S1D (I, batch 2) with their respective LiCor total protein stains (B, D, F, J, K) or β-actin loading control (L) used to ensure equal protein loading.
