# Supplementary material for: The synthetic TRPML1 agonist ML-SA1 rescues Alzheimer-related alterations of the endosomal-autophagic-lysosomal system
Source: J Cell Sci. 2023 Mar 21;136(6):jcs259875. doi: 10.1242/jcs.259875 (PMC10112969; doi:10.1242/jcs.259875)
Supplement: Supplementary file 1 [file joces-136-259875_review_history.pdf]

The synthetic TRPML1 agonist ML-SA1 rescues Alzheimer-related alterations of the endosomal-autophagic-lysosomal system

Aleksandra Somogyi, Emily D. Kirkham, Emyr Lloyd-Evans, Jincy Winston, Nicholas D. Allen, John Mackrill, Karen E. Anderson, Phillip Thomas Hawkins, Sian E. Gardiner, Helen Waller-Evans, Rebecca Sims, Barry Boland and Cora O'Neill
DOI: 10.1242/jcs.259875

Editor: Giampietro Schiavo

Review timeline

Original submission:	6 February 2022
Editorial decision:	21 March 2022
Rebuttal Received:	30 March 2022
Editorial decision:	16 May 2022
First revision received:	14 November 2022
Editorial decision:	22 December 2022
Second revision received:	3 February 2023
Accepted:	13 February 2023

Original submission

First decision letter

MS ID#: JOCES/2022/259875

MS TITLE: The synthetic TRPML1 agonist, ML-SA1, rescues Alzheimer-related alterations of the endosomal-autophagic-lysosomal system

AUTHORS: Aleksandra Somogyi, Emily D. Kirkham, Emyr Lloyd-Evans, Jincy Winston, Nicholas D. Allen, John Mackrill, Karen E. Anderson, Phillip Thomas Hawkins, Rebecca Sims, Barry Boland, and Cora O'Neill
ARTICLE TYPE: Research Article

Dear Prof O'Neill,

We have now reached a decision on the above manuscript.

To see the reviewers' reports and a copy of this decision letter, please go to:

https://submit-jcs.biologists.org and click on the 'Manuscripts with Decisions' queue in the Author Area.

(The corresponding author only has access)

As you will see from their reports, the reviewers raise a number of substantial criticisms that prevent me from accepting your paper for publication.

Clearly, the tone of the two reviewers and their support for the publication of your work are very different. After careful consideration of the information provided by your experiments, the novelty of the conclusions and the additional work required, I came to the conclusion that this manuscript does not reach sufficient priority for further consideration at JCS.

© 2023. Published by The Company of Biologists under the terms of the Creative Commons Attribution License (https://creativecommons.org/licenses/by/4.0/).

I am very sorry to give you such disappointing news, but we are currently under great pressure for space and it takes a very enthusiastic recommendation by the referees for a manuscript to be accepted.

I do hope you find the comments of the reviewers helpful in allowing you to revise the manuscript for submission elsewhere, and many thanks for sending your work to Journal of Cell Science.

Reviewer 1 Advance Summary and Potential Significance to Field:

The study by Somogy and colleagues provides evidence that abnormalities in the EAL system may be caused by dysfunction of TRPML1, a divalent cation channel on the endolysosomal membrane, and thus, activators of TRPML1 may be therapeutic against the EAL pathologies in AD. The authors used 1) AD brains, 2) human iPSC neurons (APOE manupulated), and 3) primary rat cortical neurons. In AD brains, they showed that LAMP-1 (+) vesicles or vacuoles were increased in hippocampal neurons, concomitantly with increases in PIP2 and PIP3 levels. In iPSC neuron experiments, ApoEe4 expressing ones, compared to other neurons, exhibit increases in endolysosomal calcium, the release of which by BafA1 fails to respond to TRPML1 inhibition. Combined together, this study provides a stronger evidence that TRPML1 dysfunction may play a role in the EAL pathologies observed in AD brains.
FInally in rat neurons,

Reviewer 1 Comments for the Author:
1. Fig. 1: LAMP-1 vacuoles: How was "vacuole" defined? Different from vesicles? Is vacuole the same as "granulovacuolar degeneration"? Simple histological staining may help. Western blots (D and H) seem too variable. Any thought for the reason?

2. Fig. 2: If PIP2 is increased yet TRPML1 activity is reduced, would TRPML1 agonist such as ML-SA1 (Fig 6) have the therapeutic effects? What may be the mechanism of TRPML1 hypoactivity in the presence of more PIP2, the only endogenous agonist? It seems necessary to examine the level of TRPLM1 and/or PIKfyve.

3. Fig.3: first of all, TRPML1 is permeable to not only calcium but also zinc, another divalent cation enriched in lysosomes. Furthermore, Fura2 signals sometimes are due to zinc increases (e.g. Haase et al., Cell Calcium, 2009). Hence TPEN, a specific zinc chelator, should be used as control.

Reviewer 2 Advance Summary and Potential Significance to Field:

The examination of endo-lysosomal Ca2+, APOE iPSC derived neurons are all very relevant given the need to better understand mechanisms underlying Alzheimer's disease (AD) pathology. However, this study does not provide new insight beyond prior studies (from the point of TRPML linked Ca2+ changes and APOE or with changes in endo-lysosomes on PIKfyve inhibition).

I do not feel this manuscript meets the 'Aim and scope' of JCS that a new manuscript should bring novel insight, provide significant advancement to our understanding of cell biology.

Reviewer 2 Comments for the Author:

In this study, the authors examine the effect of APOE genotype on lysosomal calcium levels and their link to TRMPL function. Based on their observations they suggest that APOE4 , an isoform associated with increased risk for AD has higher levels of endosomal Ca2+ and unable to release Ca2+ in response to TRPML activation. The authors posit firstly that these APOE4 neurons are a model for late onset AD (LOAD). The effect of APOE genotype on lysosomal Ca2+ and link to TRPML has been demonstrated before, albeit in primary cells (32517777) as are the endo-lysosomal changes on PIKfyve inhibition. Likewise, changes to endo-lysosomes upon PIKfyve inhibition have been demonstrated in several systems before. Similarly, ability of ML-SA1 to rescue enlarged endo-lysosomal phenotype has also been demonstrated before (28360104). Several conditions including activation of Rab5 cause enlargement of early endosomes. Given this, the connection between PIKfyve inhibition and AD seems tenuous. At the very least, under these conditions, Tau, Abeta

© 2023. Published by The Company of Biologists under the terms of the Creative Commons Attribution License (https://creativecommons.org/licenses/by/4.0/).

peptide levels or other read-outs more directly linked to AD should be evaluated under these conditions. Without these read-outs, the statement "ML-SA1… protects against multiple AD-related EAL neuropathologies (lines 588-590) isn't well supported. The rescue of endosomal enlargement, as mentioned above has already been demonstrated.

Additional concerns on specific figures:

1.In Fig 1A: The "perinuclear" clustered lysosome authors are examining in AD panels are in fact, lysosomes in glia. This is obvious from morphology of those cells visible even with just LAMP1 staining One can also appreciate their difference from the much larger neuronal cell bodies in Control. A couple of glia visible in this image too. It is more likely the authors have observed the increased astrogliosis in AD tissue. Colocalization with GFAP and iba1 will be needed to distinguish between lysosomal changes in CA1 neurons versus glia in that region.
2.In Figure 1E, F it is unclear what the data points are -mean intensity from 1 neuron? Are all 10 patient samples and controls being looked at here? From the graph and brief description of the statistics, it is unclear what the mean, error bars plotted here are: ideally, mean from multiple neurons per sample should be compared.
3.Fig 1D: the western blot for LAMP1 normalized with the protein stain is not convincing-one could argue that the protein levels (especially when comparing dark band at 50kDa) is increased in many of the AD samples compared to control (reflected in lack of significance in their quantification).
4.The observation that LAMP1 immunoreactivity is enriched in neuritic plaques is well established (Condello et al, 2011, Scientific Reports, Nixon et al, 2005 Gowrishankar et al, PNAS, 2015, Kandelepas et al 2016). The results describing this (Line 335) should cite prior work on this observation

---

Rebuttal letter

Dear Prof Schiavo,

Thank you for your email and decision on our manuscript. We were disappointed that, after careful consideration, our manuscript was not accepted for publication.

As you mention, the tone of the two reviewers and their support for the publication are very different. We appreciate the enthusiastic support for our manuscript by reviewer one and realise that it is preferable to receive an enthusiastic recommendation from both and not just one reviewer. However, we have serious concerns about the statements, and the supporting references used by the second reviewer to claim our work “does not provide new insight beyond prior studies”. These statements and supporting references are unfortunately not accurate, and therefore extremely misleading. From an ethical standpoint in reviewing, we believe it is important to draw your attention to this. We are requesting that our manuscript be considered by a third reviewer due to significant inaccuracies in the second review. We appreciate that JCS is committed to maintaining the production of very high-quality publications, and that this process involves a fair, constructive and efficient editorial process.

Therefore, we hope that our response to each reviewer’s comment, as outlined below, will justify a case for our manuscript to be reconsidered more favourably.

We look forward to hearing back from you and value your time and consideration of this.

Best regards,

Cora O’Neill
on behalf of the authors

Prof. Cora O’Neill
School of Biochemistry and Cell Biology

© 2023. Published by The Company of Biologists under the terms of the Creative Commons Attribution License (https://creativecommons.org/licenses/by/4.0/).

Director Cork Neuroscience C entre (CNSC)
Funded Investigator APC Microbiome Ireland
BioSciences Institute Rm 3.07
University College Cork
Cork, Ireland
Phone: 353 86 1714996
c.oneill@ucc.ie

Reviewer 1
We appreciate the enthusiastic support of our manuscript by reviewer 1. The three comments are extremely helpful, insightful and easy to respond to, as we have done in the section at the end of this email. The first paragraph of Reviewer 1's text is incomplete, it ends abruptly 'Finally in rat neurons.. Perhaps, this occurred in error, but it would be beneficial to have the complete review.

Reviewer 2

1.One of our main concerns from reviewer 2 is that *"our study does not provide new insight beyond prior studies from the point of TRPML linked Ca^{2+} changes and APOE". To support this the reviewer states "The effect of APOE genotype on lysosomal Ca2+ and link to TRPML has been demonstrated before albeit in primary cells (32517777)"*

A: This paper -32517777- (Larramona-Arcase et al., Mol Neurodegeneration 2020) from Roser Masgrau's group, is work we know well. It is performed in immortalised astrocytes, not in iPSC derived APOE cortical neurons, as we employ. The paper only compares ApoE3 and ApoE4, and is mainly about ATP-mediated Ca^{2+} release. There are two figures in the paper describing lysosomal Ca^{2+} data, where GPN was used to release Ca^{2+} stores. However, this does not have any mechanistic correlation with experiments conducted in our study, which utilised MLSA1 as a TRPML1 agonist, or in fact to any Ca^{2+} release channels (they also did not use NAADP-AM). The authors make connections to lipids instead, by looking at cholesterol. In addition, the measures of lysosomal Ca^{2+} in this paper could in fact be masked by ER Ca^{2+} as they did not clamp with ionomycin before adding GPN. It is known from recent work from Colin Taylor's group that not clamping with ionomycin definitely leads to ER store release (Atakpa et al., J. Cell Sci 2019 132 (3), so arguably, findings relating to lysosomal Ca^{2+} from Larramona-Arcase et al, may not be exclusively derived from lysosomes.

To re-iterate, there is absolutely no evidence of TRPML1-mediated Ca^{2+} release in the Larramona-Arcase et al, paper, which reviewer 2 cites as an example of our findings being shown previously. Thus, the effect of APOE genotype on lysosomal Ca^{2+} and link to TRPML1 has definitively not been demonstrated before - we have done so in this manuscript. MLSA1 and its analogues are the only agonists of TRPML1 available, a simple search of the paper for the word indicates no utilisation or mention of it in this study. TRPML1 levels are examined by qPCR, but that is meaningless if channel activation is not measured.

2.The second main concern of the reviewer is that:
"Likewise, changes to endo-lysosomes upon PIKfyve inhibition have been demonstrated in several systems before. Similarly, ability of ML-SA1 to rescue enlarged endo-lysosomal phenotype has also been demonstrated before (28360104)".

A: It is true that changes in endolysosomes upon PIKfyve inhibition have been demonstrated in several cell systems before. However, there has been very little demonstration of this in neuronal systems. Moreover, the degree of endolysosomal change we discovered to be induced by PIKfyve inhibition in primary neurons (increased endolysosomal Ca^{2+} accumulation, enlargement and perinuclear clustering of endolysosomes, autophagic vesicle accumulation, and early endosomal enlargement) has never been demonstrated before. So, to reiterate further this finding described in our work is novel and has not been demonstrated before and importantly recapitulates the EAL (endosomal-autophagic lysosomal) neuropathology of AD.

The paper that is cited to support this second main concern of the reviewer "*Similarly, ability of ML-SA1 to rescue enlarged endo-lysosomal phenotype has also been demonstrated before (28360104)*. This paper by Cao et al., JBC is entitled "The lysosomal calcium release channel

© 2023. Published by The Company of Biologists under the terms of the Creative Commons Attribution License (https://creativecommons.org/licenses/by/4.0/).

TRPML1 regulates lysosomal size by activating calmodulin". The Cao et al., study is performed in COS-1 cells, not primary neurons, and has very little connection to neurons, AD or APOE. They simply show ML-SA1 rescues enlargement of endolysosomes in Cos-1 cells. In our study, we show that the multiple EAL defects we discovered in neurons (increased endolysosomal Ca^{2+} accumulation, enlargement and perinuclear clustering of endolysosomes, autophagic vesicle accumulation, and early endosomal enlargement), are all rescued by TRPML1 activation using ML-SA1. This finding is also novel, as it has never been demonstrated before that ML-SA1 protects against this multitude of EAL phenotypes that we discovered to be caused by PIKfyve inhibition in neurons. These results further indicate for the first time that targeting TRMPL1 protects against multiple EAL neuropathologies that occur in AD.

To reiterate, the papers the reviewer uses to support statements that our work "does not provide new insight beyond prior studies" do not support these statements at all. The very definitive statements made by the reviewer in this respect are unfortunately extremely misleading and not supported by the references cited which is a major concern to us.

All of the other comments and suggestions of reviewer 2 are constructive and easy to respond to, as we have outlined below.

3. Several conditions including activation of Rab5 cause enlargement of early endosomes. Given this, the connection between PIKfyve inhibition and AD seems tenuous.

A: Yes, several conditions, including activation of Rab5, cause enlargement of early endosomes in neurons. However, very few, if any, conditions that we are aware of cause enlargement of early endosomes, endolysosomal Ca^{2+} accumulation, enlargement and perinuclear clustering of endolysosomes, and autophagic vesicle accumulation as we report in this study with the inhibition of PIKfyve. All of these pathological EAL phenotypes are evident in neurons in the AD brain and many of them are evident in APOE4 neurons. Therefore, we believe it surely is an important discovery that inhibiting PIKfyve can induce multiple AD pathologies while activation of RAB5 cannot. We thank the reviewer for bringing attention to this, as we may have failed in explaining the significance and novelty of these findings in sufficient detail.

4. *At the very least, under these conditions, Tau, Abeta peptide levels or other read-outs more directly linked to AD should be evaluated under these conditions. Without these read-outs, the statement "ML-SA1… protects against multiple AD-related EAL neuropathologies (lines 588-590) isn't well supported"*

A: We respectively disagree that the statement *"ML-SA1… protects against multiple AD-related EAL neuropathologies (lines 588-590) isn't well supported"*. As we explained in sections above, ML-SA1 does protect against multiple AD related EAL neuropathologies and we show this for the first time. We agree that measurement of tau and Abeta peptide levels would add further. However, our discovery that PIKfyve inhibition can induce these EAL neuropathologies which are protected against by TRPML1 activation does stand on its own. Notably, Cataldo and colleagues showed that endocytic pathways abnormalities precede amyloid beta deposition in late onset AD and in Down's syndrome (Cataldo AM et a., Amer. J. Pathol 2000, 157, 277-286)

Reviewer 2 Additional concerns on specific figures:

1.In Fig 1A: The "perinuclear" clustered lysosome authors are examining in AD panels are in fact, lysosomes in glia. This is obvious from morphology of those cells visible even with just LAMP1 staining One can also appreciate their difference from the much larger neuronal cell bodies in Control. A couple of glia visible in this image too. It is more likely the authors have observed the increased astrogliosis in AD tissue. Colocalization with GFAP and iba1 will be needed to distinguish between lysosomal changes in CA1 neurons versus glia in that region.

A: We agree with the reviewer that colocalization of LAMP1 with GFAP and Iba1 will be needed to distinguish between lysosomal changes in CA1 neurons versus glia. We respectively disagree that the perinuclear clustering of lysosomes in the AD patients are in fact lysosomes in glia. The 3D stack rendering on the enlarged cells in Figure 1 are definitely neurons, the nuclei and cells are the same size in the control and AD section and the perinuclear clustering of lysosomes AD neurons is

© 2023. Published by The Company of Biologists under the terms of the Creative Commons Attribution License (https://creativecommons.org/licenses/by/4.0/).

highly evident here. Likewise, the CA3 cells with enlarged LAMP1 decorated vacuoles are neurons. It is possible that some glia also have this phenotype which would be clarified by glial and neuronal markers. Notably, Cataldo et al, report distinct accumulation of cathepsin-D-positive lysosomes in neurons in AD brain sections (Cataldo AM et al., Neurons, 1995, 14, 678-80)

2.In Figure 1E, F it is unclear what the data points are -mean intensity from 1 neuron? Are all 10 patient samples and controls being looked at here? From the graph and brief description of the statistics, it is unclear what the mean, error bars plotted here are: ideally, mean from multiple neurons per sample should be compared.

A: We apologise that this is unclear. The data presented in Fig 1E, F is from n = 6 AD cases and n = 8 control cases with 2-5 representative images analysed for each case, together analysing n = 26 control images and n = 26 AD images.

3.Fig 1D: the western blot for LAMP1 normalized with the protein stain is not convincing-one could argue that the protein levels (especially when comparing dark band at 50kDa) is increased in many of the AD samples compared to control (reflected in lack of significance in their quantification).

A: We agree that western blot Fig 1D are variable. However, one can see that the total protein stain verifies equality of protein loading of each of the control and AD cases. This are the levels of LAMP1 present in the control and AD cases. This blot was repeated to yield this consistent result. It most likely arises from actual variability in LAMP1 between control and AD cases, although many of the AD cases do have high levels of LAMP1, as pointed out by the reviewer, this did not attain significance due to the variability in LAMP1 levels when comparing the total number of AD and control cases. We would be happy to include further analysis of LAMP1 in a larger number of control and AD cases to clarify this issue. The result may also reflect the mixture of cell types that are present in such human brain lysates

4.The observation that LAMP1 immunoreactivity is enriched in neuritic plaques is well established (Condello et al, 2011, Scientific Reports, Nixon et al, 2005 Gowrishankar et al, PNAS, 2015, Kandelepas et al 2016). The results describing this (Line 335) should cite prior work on this observation

A: Yes, this is indeed well established, and these papers need to be cited, we thank the reviewer for pointing out this omission.

For reviewer 1, the questions are very easily addressed as outlined below:

1. Fig. 1: LAMP-1 vacuoles: How was "vacuole" defined? Different from vesicles? Is vacuole the same as "granulovacuolar degeneration"? Simple histological staining may help. Western blots (D and H) seem too variable. Any thought for the reason?

A: The vacuole was defined as a large membrane bound vacuole that was immunoreactive to LAMP1. These vacuoles were larger than vesicles, approx. 4-10mM diameter. In granulovacuolar degeneration the vacuoles have a dense central core or central granule, which was not evident in the vacuoles we show. However, it would be important to verify this. As the reviewer states, this can be easily achieved using the specific markers for the granulovacuolar granule, CKIdelta (Funk, Mrak and Kuret 2011) or CHMP2B, and would be very informative.

We agree that western blot Fig 1D seem variable. However, one can see that the total protein stain verifies equality of protein loading and this are the levels of LAMP1 present in control and AD cases. This blot was repeated to yield this consistent result. It most likely arises from actual variability in LAMP1 between control and AD cases although many of the AD cases do have high levels of LAMP1. We would be happy to include further analysis of LAMP1 in a larger number of control and AD cases to clarify this issue. The result may also reflect the mixture of cell types that are present in such human brain lysates

2. Fig. 2: If PIP2 is increased yet TRPML1 activity is reduced, would TRPML1 agonist such as ML-SA1 (Fig 6) have the therapeutic effects? What may be the mechanism of TRPML1 hyperactivity in the

© 2023. Published by The Company of Biologists under the terms of the Creative Commons Attribution License (https://creativecommons.org/licenses/by/4.0/).

presence of more PIP2, the only endogenous agonist? It seems necessary to examine the level of TRPLM1 and/or PIKfyve.

A: Although, levels of $PI(4,5)P_2$ are increased, it is still not possible to measure levels of the stereoisomer $PI(3,5)P_2$, which is the endogenous TRPML1 agonist. It would indeed add to examine the levels of TRPML1 and/or components of the PIKfyve kinase complex. We would also like to point out that MLSA1 'locks' the TRPML1 channel in an open conformation, so it will work regardless of the levels of $PI(4,5)P_2$ (antagonist) or $PI(3,5)P_2$ (agonist). This is best evidenced by a study of Zou et al., (J. Neurosci (2015) 35, 6801-12) on Fig4 deficiency. Fig4 prevents the synthesis of $PI(3,5)P_{2,}$ where TRPML1 activity is also reduced and MLSA1 was shown rescue this Fig4 deficiency and activate TRPML1. We do have data investigating TRPML1 levels and localization in AD and control cases, but as TRPML1 antibodies have been criticised due to possible non-specificity we did not include it. We would be happy to provide data on TRPML1 levels. In addition, we have preliminary data for components of the PIKfyve complex, which could be applied to analyse control and AD cases.

3. Fig.3: first of all, TRPML1 is permeable to not only calcium but also zinc, another divalent cation enriched in lysosomes. Furthermore, Fura2 signals sometimes are due to zinc increases (e.g. Haase et al., Cell Calcium, 2009). Hence TPEN, a specific zinc chelator, should be used as control.

A: TRPML1 is permeant to $Zn^{2+,}$ but only at microM concentrations, which are not physiological (Zn^{2+} levels in cells are low nM), if levels ever reach mM then it damages membranes, including lysosomes, as shown in Kobayashi et al, (Nat Cell Biol 1999). The zinc accumulation in TRPML1 deficient MLIV patient cells are only observed when the cells are grown in extracellular medium supplemented with 100 mM zinc (as outlined in many publications by Kirill Kiselyov's papers for example), which is non-physiological.

Original submission

First decision letter

MS ID#: JOCES/2022/259875

MS TITLE: The synthetic TRPML1 agonist, ML-SA1, rescues Alzheimer-related alterations of the endosomal-autophagic-lysosomal system

AUTHORS: Aleksandra Somogyi, Emily D. Kirkham, Emyr Lloyd-Evans, Jincy Winston, Nicholas D. Allen, John Mackrill, Karen E. Anderson, Phillip Thomas Hawkins, Rebecca Sims, Barry Boland, and Cora O'Neill
ARTICLE TYPE: Research Article

We have now reached a decision on the above manuscript.

Based on your rebuttal letter and the correspondence that followed, we were able to secure two additional reviewers, which confirmed the potential interest of the data included in the manuscript, but at the same time highlighted several weaknesses in the current version of the manuscript.

In contrast to my previous views, albeit editorial and experimental work are needed to fix these problems, I now feel that a fully revised version addressing these concerns might prove acceptable. If you think that you can deal satisfactorily with the criticisms on revision (including adding at least part of the data not shown in the supplemental material), I would be pleased to see a revised manuscript. We would then return it to the four reviewers.

To see the reviewers' reports and a copy of this decision letter, please go to: https://submit-jcs.biologists.org and click on the 'Manuscripts with Decisions' queue in the Author Area. (Corresponding author only has access to reviews.)you

© 2023. Published by The Company of Biologists under the terms of the Creative Commons Attribution License (https://creativecommons.org/licenses/by/4.0/).

Please ensure that you clearly highlight all changes made in the revised manuscript. Please avoid using 'Tracked changes' in Word files as these are lost in PDF conversion.

I should be grateful if you would also provide a point-by-point response detailing how you have dealt with the points raised by the reviewers in the 'Response to Reviewers' box. Please attend to all of the reviewers' comments. If you do not agree with any of their criticisms or suggestions please explain clearly why this is so.

Reviewer 1

Advance summary and potential significance to field

The study by Somogy and colleagues provides evidence that abnormalities in the EAL system may be caused by dysfunction of TRPML1, a divalent cation channel on the endolysosomal membrane, and thus, activators of TRPML1 may be therapeutic against the EAL pathologies in AD. The authors used 1) AD brains, 2) human iPSC neurons (APOE manupulated), and 3) primary rat cortical neurons. In AD brains they showed that LAMP-1 (+) vesicles or vacuoles were increased in hippocampal neurons, concomitantly with increases in PIP2 and PIP3 levels. In iPSC neuron experiments, ApoEe4 expressing ones, compared to other neurons, exhibit increases in endolysosomal calcium, the release of which by BafA1 fails to respond to TRPML1 inhibition. Combined together, this study provides a stronger evidence that TRPML1 dysfunction may play a role in the EAL pathologies observed in AD brains.
FInally in rat neurons,

Comments for the author

1. Fig. 1: LAMP-1 vacuoles: How was "vacuole" defined? Different from vesicles?
Is vacuole the same as "granulovacuolar degeneration"? Simple histological staining may help.
Western blots (D and H) seem too variable. Any thought for the reason?

2. Fig. 2: If PIP2 is increased yet TRPML1 activity is reduced, would TRPML1 agonist such as ML-SA1 (Fig 6) have the therapeutic effects? What may be the mechanism of TRPML1 hypoactivity in the presence of more PIP2, the only endogenous agonist? It seems necessary to examine the level of TRPLM1 and/or PIKfyve.

3. Fig.3: first of all, TRPML1 is permeable to not only calcium but also zinc another divalent cation enriched in lysosomes. Furthermore, Fura2 signals sometimes are due to zinc increases (e.g. Haase et al., Cell Calcium, 2009). Hence TPEN, a specific zinc chelator, should be used as control.

Reviewer 2

Advance summary and potential significance to field

The examination of endo-lysosomal Ca2+, APOE iPSC derived neurons are all very relevant given the need to better understand mechanisms underlying Alzheimer's disease (AD) pathology. However, this study does not provide new insight beyond prior studies (from the point of TRPML linked Ca2+ changes and APOE or with changes in endo-lysosomes on PIKfyve inhibition).

I do not feel this manuscript meets the 'Aim and scope' of JCS that a new manuscript should bring novel insight, provide significant advancement to our understanding of cell biology.

Comments for the author

In this study, the authors examine the effect of APOE genotype on lysosomal calcium levels and their link to TRMPL function. Based on their observations they suggest that APOE4 , an isoform associated with increased risk for AD has higher levels of endosomal Ca2+ and unable to release Ca2+ in response to TRPML activation.
The authors posit firstly that these APOE4 neurons are a model for late onset AD (LOAD). The effect of APOE genotype on lysosomal Ca2+ and link to TRPML has been demonstrated before, albeit in

© 2023. Published by The Company of Biologists under the terms of the Creative Commons Attribution License (https://creativecommons.org/licenses/by/4.0/).

primary cells (32517777) as are the endo-lysosomal changes on PIKfyve inhibition. Likewise, changes to endo-lysosomes upon PIKfyve inhibition have been demonstrated in several systems before. Similarly, ability of ML-SA1 to rescue enlarged endo-lysosomal phenotype has also been demonstrated before (28360104). Several conditions including activation of Rab5 cause enlargement of early endosomes. Given this, the connection between PIKfyve inhibition and AD seems tenuous. At the very least, under these conditions, Tau, Abeta peptide levels or other read-outs more directly linked to AD should be evaluated under these conditions.
Without these read-outs, the statement “ML-SA1… protects against multiple AD-related EAL neuropathologies (lines 588-590) isn’t well supported. The rescue of endosomal enlargement, as mentioned above has already been demonstrated.

Additional concerns on specific figures:

1. In Fig 1A: The “perinuclear” clustered lysosome authors are examining in AD panels are in fact lysosomes in glia. This is obvious from morphology of those cells visible even with just LAMP1 staining One can also appreciate their difference from the much larger neuronal cell bodies in Control. A couple of glia visible in this image too. It is more likely the authors have observed the increased astrogliosis in AD tissue.
Colocalization with GFAP and iba1 will be needed to distinguish between lysosomal changes in CA1 neurons versus glia in that region.
2. In Figure 1E, F it is unclear what the data points are -mean intensity from 1 neuron? Are all 10 patient samples and controls being looked at here? From the graph and brief description of the statistics, it is unclear what the mean, error bars plotted here are: ideally, mean from multiple neurons per sample should be compared.
3. Fig 1D: the western blot for LAMP1 normalized with the protein stain is not convincing-one could argue that the protein levels (especially when comparing dark band at 50kDa) is increased in many of the AD samples compared to control (reflected in lack of significance in their quantification).
4. The observation that LAMP1 immunoreactivity is enriched in neuritic plaques is well established (Condello et al, 2011, Scientific Reports, Nixon et al, 2005 Gowrishankar et al, PNAS, 2015, Kandelepas et al 2016). The results describing this (Line 335) should cite prior work on this observation

Reviewer 3

Advance summary and potential significance to field

This is a timely manuscript by an excellent group with substantial experience in the calcium and AD fields. The greater lysosomal system is rather under-appreciated in the AD field and therefore this manuscript helps highlight its importance. The writing was excellent, the experiments appear to have been conducted appropriately, and their findings would be an addition to the field. However, I do have some points that once addressed might make this manuscript even stronger.

Comments for the author

The authors either missed or chose not to include substantial literature showing that ML-SA1 protects against Alzheimer’s-like pathology that occurs in other neurological conditions including HIV-1 associated neurocognitive disorder. They might start with the following publication in which it was shown that ML-SA1 promoted calcium efflux, luminal acidification, and cleared both sphingomyelin and A*β* from lysosomes.

Bae, M., Patel, N. Xu, H., Lee, M., Nath, A., Geiger, J.D., Gorospe, M.M., Mattson, M.P., and Haughey, N.J. (2014) Activation of TRPML1 Clears Intraneuronal A*β* in Pre-Clinical Models of HIV-Infection. J. Neurosci. 34 (34): 11485-11503.

There is also substantial literature on how insults related to AD pathogenesis cause increases in pH of endosomes and lysosomes and how ML-SA1 can rescue not only the pH but also AD-like pathological effects.

© 2023. Published by The Company of Biologists under the terms of the Creative Commons Attribution License (https://creativecommons.org/licenses/by/4.0/).

Therefore, I recommend either measuring vacuolar pH or at the very least addressing these issues in the manuscript. Especially because calcium accumulation, perinuclear clustering, and effects on organellar volume are affected by vacuolar pH.
They were singularly focused on the divalent cation calcium. However, endosomes and lysosomes also contain other divalent cations, the release of which is also influenced by pH and TRPML channels. Therefore they should at least comment on their possible involvement in the observed subcellular changes as they relate to AD.

Calcium release experiments were performed with GPN and bafilomycin and showed effects on lysosome calcium levels and release. They could have introduced or commented on possible inter-organellar signaling by the released calcium. They showed changes in relative levels of calcium in lysosomes but not actual levels (probably in the hundreds of micromolar). If they have those data I think they should be included.

Phosphoinositides are very labile substances. Others and they could not measure levels of the major endogenous agonist of TRPML channels PI(3,5)P2. It might be helpful to know what the postmortem intervals for the human brain samples were and whether there was any correlation between PI levels and post mortem intervals.

Minor issues:

In the Results section they need to refer to their data as 'these data' not "this data".

Reviewer 4

Advance summary and potential significance to field

The authors present a thorough evaluation of the contribution of TRPML1 to LOAD phenotype in patient brain sections, APOE iPSC-neurons and rat primary neurons.
They show that in LOAD post-mortem CA1/CA3 brain sections that LAMP1+ vesicles cluster in perinuclear endolysosomes (EL), APOE4 iPSC cortical neurons have decreased TRPML1-induced EL Ca2+ release compared to other APOE allelic isoforms and pharmacological inhibition of TRPML1 activation via PIKfyve-PIP3 recapitulated LOAD phenotype in primary neurons. This phenotype was rescued using ML-SA1 agonist. Confirming the mechanistic link between PIKfyve-PI(3,5)P2-TRPML1 in LOAD neurons has not been done before which makes this manuscript novel and provides significant new information to the field relating EL vesicular transport and more specifically TRPML1 to Alzheimer's disease pathobiology.

Comments for the author

Overall, the manuscript is very well written apart from the continued fact that the figures and sub-figure orders do not follow the chronology of the Results body text. This culminates in the final two Results sections flipping back and forth between Figures 5 and 6. I think this is easily resolved and needs to be altered.
Finally, I see no reason why all the data referred to as "data not shown" should not be shown, some of it is important in terms of reference or substantiating other datasets. I would recommend much of it is presented in Supplementary Data.

Major Comments
1. ICC is conducted using LAMP1 in brain sections and iPSC-neurons but Rab7 in rat primary neurons to label EL. It could be argued that neither are bona fide markers of EL, with LAMP1 being a mature lysosome marker and Rab7 being implicated in many aspects of membrane trafficking in the late endocytic and autophagic network. This may require more specific classifications. This comment also cross-references to comment 6.
2. It is disappointing that PI(3,5)P2 cannot be detected on mass spec somewhat diminishing the link between PI(3,5)P2 and TRPML1. Mass spec analyses of phosphoinositol species in LOAD patients compared to age-matched controls shows no significant difference in PI or total PIP and a significant increase in PIP2 and PIP3. However, only the increase in PIP2 is discussed in terms of relevance. This requires an expanded justification in the Results section.

© 2023. Published by The Company of Biologists under the terms of the Creative Commons Attribution License (https://creativecommons.org/licenses/by/4.0/).

3. The data showing that APOE4 EL load elevated Ca2+ and that release is compromised through pH-dependent TRPML1 activity is compelling and well presented in iPSC-derived neurons.

4. The ability of ML-SA1 to rectify TRPML1 activity following PIKfyve inhibition in rat cortical neurons is equally compelling.
5. The time and dose-responsive vacuolisation caused by YM201636 treatment in rat primary neurons is clear with Rab7. As the authours describe this is not seen with Rab5, EEA1 and LBPA I think it would be equally important to show that it also occurs with LAMP1 even if this is only conducted at 24h with 4μM of YM201636.
This would provide consistency in vacuolisation between the different cells analysed in the manuscript.

Minor Comments
1. Figure 1A-C. There is no evidence presented that these are neurons, the staining is LAMP1 ICC and DAPI nuclear stain.
2. Figure 1I & J. There is no evidence presented that the stained cells are pyramidal neurons as described in the text. The staining is LAMP1 (red), PHF1
(green) ICC and DAPI nuclear stain.
3. Figure 1D shows a western blot for LAMP1 on Braak staged tissue but is mentioned much later in the text then quantified in Fig. 1H. It would make sense to me having the figures in order of textual description.
4. Figure 3A should be sub-categorised into (i) and (ii). 3C should precede 3B in my mind as these are some of the data shown in B. Why not show all the traces i.e. with and without inhibitor (which is described as GW405833 in the body text but ML-SI1 in the figure.
5. Fig. 3. The data for positive activation of TRPML1 with ML-SA1 should be presented, even if it is included as Supplementary Data.
6. Fig. 3. It would be good to see the Ca spark Fura-2 profiles with as well as without TRPML1 inhibitor.
7. Fig. 4. The data for YM201636 inhibition of PIKfyve at 6h should be presented in Supp Data. Conversely, data in Fig. 4C could go into supps as 4B shows the reversing effect of ML-SA1.
8. Fig. 5. Rab5, EEA1 and LBPA ICC should be presented in Supps. It is relevant to show proof that this effect is seen specifically with Rab7.

First revision

Author response to reviewers' comments

Response to Reviewers

We have thoughtfully taken the comments of the reviewers into account and addressed all the concerns raised. The required additional experimental data have been incorporated into the revised manuscript and the appropriate revisions made. We have included all information detailing our response to reviewers' comments in the associated manuscript and response below. We thank the reviewers for their review and for their insightful comments and believe that addressing the important points raised has significantly strengthened the significance of the study.

Reviewer 1

Advance Summary and Potential Significance to Field:

The study by Somogyi and colleagues provides evidence that abnormalities in the EAL system may be caused by dysfunction of TRPML1, a divalent cation channel on the endolysosomal membrane, and thus, activators of TRPML1 may be therapeutic against the EAL pathologies in AD. The authors used 1) AD brains, 2) human iPSC neurons (APOE manipulated), and 3) primary rat cortical neurons. In AD brains, they showed that LAMP-1 (+) vesicles or vacuoles were increased in hippocampal neurons, concomitantly with increases in PIP2 and PIP3 levels. In iPSC neuron experiments, ApoEe4 expressing ones, compared to other neurons, exhibit increases in

© 2023. Published by The Company of Biologists under the terms of the Creative Commons Attribution License (https://creativecommons.org/licenses/by/4.0/).

endolysosomal calcium, the release of which by BafA1 fails to respond to TRPML1 inhibition. Combined together, this study provides a stronger evidence that TRPML1 dysfunction may play a role in the EAL pathologies observed in AD brains.
Finally in rat neurons,

We thank the reviewer and appreciate their support of our manuscript highlighting that our study provides strong evidence that TRPML1 dysfunction may play a role in EAL pathologies in AD brain. The comments for the author are extremely valuable, we have addressed these below, and believe that they have improved the study

Comments for the Author:

1. *Fig. 1: LAMP-1 vacuoles: How was "vacuole" defined? Different from vesicles? Is vacuole the same as "granulovacuolar degeneration"? Simple histological staining may help. Western blots (D and H) seem too variable. Any thought for the reason?*

A: The vacuole was defined as a large membrane bound vacuole that was immunoreactive to LAMP1. These vacuoles were larger than vesicles, approx. 4-10 μm in diameter. In granulovacuolar degeneration the vacuoles have a dense central core or central granule, which was not evident in the vacuoles we show. We have now included text to clarify this (page 6, lines 170-175). We also attempted to verify this, as the reviewer suggested, using a specific marker for the granulovacuolar granule, casein kinase-1 delta (CK-1δ) (Funk, Mrak and Kuret Neuropathol Appl NBiol (2011) 37: 295-306) and double immunofluorescence with LAMP1. However, immunofluorescence analysis with CK-1δ antibody did not reveal clear granulovacuolar granule staining, we have included this information in the results text (page 6/7, line 175-180).

We agree that the western blot (now Fig 1 I, J) seems variable. However, one can see that the total protein stain verifies equality of protein loading, thus these are the levels of LAMP1 present in these control and AD cases. This result most likely arises from actual variability in LAMP1 levels when comparing control and AD cases, although many of the AD cases have high levels of LAMP1 this does not attain significance. These results agree with previously published work where western immunoblot analysis also found LAMP1 levels were not significantly different when comparing brain samples from control and AD patients (Bordi et al., 2016). These results may also reflect the mixture of cell types that are present in such human brain lysates. We have included additional information in the results (Page 7, lines 191-196) to clarify this.

2. *Fig. 2: If PIP2 is increased yet TRPML1 activity is reduced, would TRPML1 agonist such as ML- SA1 (Fig 6) have the therapeutic effects? What may be the mechanism of TRPML1 hyperactivity in the presence of more PIP2, the only endogenous agonist? It seems necessary to examine the level of TRPLM1 and/or PIKfyve.*

A: Although levels of the TRPML1 antagonist, PI(4,5)P2, are increased, it is still not possible to measure levels of the stereoisomer PI(3,5)P2, which is the endogenous TRPML1 agonist. We would also like to point out that ML-SA1 'locks' the TRPML1 channel in an open conformation, so it will work regardless of the levels of PI(4,5)P2 (antagonist) or PI(3,5)P2 (agonist). This is best evidenced by a study of Zou *et al.*, (J. Neurosci (2015) 35, 6801-12) on FIG4 deficiency. FIG4 prevents the synthesis of PI(3,5)P2, resulting in reduced TRPML1 activity and ML-SA1 was shown to activate TRPML1 and rescue this FIG4 deficiency. .

It would indeed add to examine the levels of TRPML1 and/or components of the PIKfyve kinase complex. We have data investigating TRPML1 levels in AD and control cases.
However, as TRPML1 antibodies have been criticised due to possible non-specificity and have not been characterised in systems in which TRPML1 is deleted, we had not included this. We found variability in antigenicity and immunoblot profiles between TRPML1 antibody batches from the same commercial source (Figure S1B-D). Nevertheless, we have now included our data investigating TRPML1 levels in control and AD samples by western immunoblot analysis (Figure S1B, C) and immunofluorescence (Figure S1D). We now clearly state this caveat regarding possible non-specificity and variability of the TRPML1 antibody in the manuscript (Page 8, line 218-225). Overall, our analysis of TRPML1 levels and localisation by western immunoblot and immunofluorescence

© 2023. Published by The Company of Biologists under the terms of the Creative Commons Attribution License (https://creativecommons.org/licenses/by/4.0/).

analysis indicate that it is the cellular localisation and of TRPML1 rather than overall levels that are altered in AD. However, this interpretation comes with a proviso regarding TRPML1 antibody specificity.

3. *Fig.3: first of all, TRPML1 is permeable to not only calcium but also zinc, another divalent cation enriched in lysosomes. Furthermore, Fura2 signals sometimes are due to zinc increases (e.g. Haase et al., Cell Calcium, 2009). Hence, TPEN, a specific zinc chelator, should be used as control.*

A: Yes, we agree with the reviewer that TRPML1 is permeable to Zn^{2+}. However, this only occurs at μM Zn^{2+} concentrations, which are not physiological (Zn^{2+} levels in cells are low nM) - if levels ever reach μM then it damages membranes, including lysosomes, as shown by Kobayashi *et al.*, (1999) Nat. Cell Biol. 1: 113-8. The zinc accumulation in TRPML1 deficient MLIV patient cells is only observed when the cells are grown in extracellular medium supplemented with 100 μM zinc (as outlined in many publications by Kirill Kiselyov's team for example), which is non-physiological. Furthermore, the acidic pH of lysosomes renders Zn^{2+} chelation by TPEN ineffective (Huang et al., 2013, Metallomics).

To address the reviewer's concern, we performed additional experiments with the *APOE* isoform-expressing iPSC-derived neurons using the FluoZin3(AM) probe to detect Zn^{2+} accumulation. Our results show that no significant difference in Zn^{2+} levels were detected when comparing the *APOE* modified iPSC-derived cortical neurons (Figure S2A) and results text (page 10, line 278-284). This is not surprising, confirming what is stated above, as Zn^{2+} accumulation in cells with a TRPML1 defect would only be observed when the cells are grown in 100 μM extracellular zinc (Minckley *et al.*, Nat. Commun. (2019) 10: 4806). We did not investigate this, as this concentration of $ZnCl_2$ is toxic to cells.

Reviewer 2

Advance Summary and Potential Significance to Field:

The examination of endo-lysosomal Ca2+, APOE iPSC derived neurons are all very relevant given the need to better understand mechanisms underlying Alzheimer's disease (AD) pathology. However, this study does not provide new insight beyond prior studies (from the point of TRPML linked Ca2+ changes and APOE or with changes in endo-lysosomes on PIKfyve inhibition).

I do not feel this manuscript meets the 'Aim and scope' of JCS that a new manuscript should bring novel insight, provide significant advancement to our understanding of cell biology.

Comments for the Author:

In this study, the authors examine the effect of APOE genotype on lysosomal calcium levels and their link to TRMPL function. Based on their observations they suggest that APOE4, an isoform associated with increased risk for AD has higher levels of endosomal Ca2+ and unable to release Ca2+ in response to TRPML activation. The authors posit firstly that these APOE4 neurons are a model for late onset AD (LOAD). The effect of APOE genotype on lysosomal Ca2+ and link to TRPML has been demonstrated before, albeit in primary cells (32517777) as are the endo-lysosomal changes on PIKfyve inhibition. Likewise, changes to endo-lysosomes upon PIKfyve inhibition have been demonstrated in several systems before. Similarly, ability of ML-SA1 to rescue enlarged endo-lysosomal phenotype has also been demonstrated before (28360104). Several conditions including activation of Rab5 cause enlargement of early endosomes. Given this, the connection between PIKfyve inhibition and AD seems tenuous. At the very least, under these conditions, Tau, Abeta peptide levels or other read-outs more directly linked to AD should be evaluated under these conditions.

Without these read-outs, the statement "ML-SA1… protects against multiple AD-related EAL *neuropathologies (lines 588-590) isn't well supported. The rescue of endosomal enlargement, as mentioned above has already been demonstrated.*

© 2023. Published by The Company of Biologists under the terms of the Creative Commons Attribution License (https://creativecommons.org/licenses/by/4.0/).

1. One of the main concerns of reviewer 2 is that *"our study does not provide new insight beyond prior studies from the point of TRPML linked Ca^{2+} changes and APOE". To support this the reviewer states "The effect of APOE genotype on lysosomal Ca2+ and link to TRPML has been demonstrated before albeit in primary cells (32517777)"*

A: This paper -32517777- (Larramona-Arcase *et al.*, Mol Neurodegeneration 2020) from Roser Masgrau's group, is work we know well. It is performed in immortalised astrocytes, not in iPSC derived *APOE* cortical neurons, as we employ. The paper only compares *APOE* ε3 and APOE ε4 and is mainly about ATP-mediated Ca^{2+} release. There are two figures in the paper describing lysosomal Ca^{2+} data, where GPN was used to release Ca^{2+} stores.
However, this does not have any mechanistic correlation with experiments conducted in our study, which utilised TRMPL1 antagonists and ML-SA1 as a TRPML1 agonist, or in fact to any Ca^{2+} release channels (they also did not use NAADP-AM). The authors make connections to lipids instead, by looking at cholesterol. In addition, the measures of lysosomal Ca^{2+} in this paper could in fact be masked by ER Ca^{2+} as they did not clamp with ionomycin before adding GPN. It is known from recent work from Colin Taylor's group that not clamping with ionomycin definitely leads to ER store release (Atakpa *et al.*, J. Cell Sci 2019 132 (3)), so arguably, findings relating to lysosomal Ca^{2+} from Larramona-Arcase *et al.*, may not be exclusively derived from lysosomes.

To reiterate, there is absolutely no evidence of TRPML1-mediated Ca^{2+} release in the Larramona-Arcase *et al.* paper, which reviewer 2 cites as an example of our findings being shown previously. Thus, the effect of *APOE* genotype on lysosomal Ca^{2+} and link to TRPML1 has definitively not been demonstrated before - we have done so in this manuscript. In addition, in this revised manuscript we provide further novel information on this, please see response to reviewer 3. ML-SA1/3/5 and ML-SI1/3 and its analogues are the only available agonists and antagonists of TRPML1 and there is no utilisation or mention of them in this study. TRPML1 levels are examined by qPCR, but that is meaningless if channel activation is not measured. To address the reviewer's concern, we have emphasised the novelty of our findings revealing defects in TRMPL1 mediated Ca^{2+} release in APOE ε4 neurons (Discussion, page 15, paragraph 2 and line 460).

2. The second main concern of the reviewer is that:
"Likewise, changes to endo-lysosomes upon PIKfyve inhibition have been demonstrated in several systems before. Similarly, ability of ML-SA1 to rescue enlarged endo-lysosomal phenotype has also been demonstrated before (28360104)".

A: It is true that changes in endolysosomes upon PIKfyve inhibition have been demonstrated in several cell systems before. However, there has been very little demonstration of this in neuronal systems. Moreover, the degree of endolysosomal change we discovered to be induced by PIKfyve inhibition in primary neurons (increased endolysosomal Ca^{2+} accumulation, enlargement and perinuclear clustering of endolysosomes, autophagic vesicle accumulation, and early endosomal enlargement) has never been demonstrated before. So, to reiterate further this finding described in our work is novel and has not been demonstrated before and importantly recapitulates the EAL (endosomal-autophagic lysosomal) neuropathology of AD. To address the reviewers concerns we have highlighted the novelty of these findings in the Discussion (page 15, line 428-437 and page 18, line 532- 537).

The paper by Cao *et al.*, JBC that is cited to support this second main concern of the reviewer *"Similarly, ability of ML-SA1 to rescue enlarged endo-lysosomal phenotype has also been demonstrated before (28360104)."* is entitled "The lysosomal calcium release channel TRPML1 regulates lysosomal size by activating calmodulin". The Cao *et al.*, study is performed in COS-1 cells, not primary neurons, and has very little connection to neurons, AD or *APOE*. They simply show ML-SA1 rescues enlargement of endolysosomes in Cos-1 cells.
In our study, we show that the multiple EAL defects we discovered in neurons (increased endolysosomal Ca^{2+} accumulation, enlargement and perinuclear clustering of endolysosomes, autophagic vesicle accumulation, and early endosomal enlargement), are all rescued by TRPML1 activation using ML-SA1. This finding is also novel, as it has never been demonstrated before that ML-SA1 protects against this multitude of EAL neuropathologies that occur in AD and that we discovered to be caused by PIKfyve inhibition in neurons. We have also emphasised the novelty of these findings in the discussion (page 15, line 428-437 and page 18, line 532-537).

© 2023. Published by The Company of Biologists under the terms of the Creative Commons Attribution License (https://creativecommons.org/licenses/by/4.0/).

To reiterate, the papers the reviewer uses to support statements that our work “does not provide new insight beyond prior studies” do not support these statements at all. The definitive statements made by the reviewer in this respect are unfortunately extremely misleading and not supported by the references cited, which is a major concern to us. We have thus amended the discussion to highlight how our work provides new insight beyond prior studies as outlined above, and as highlighted by the other 3 reviewers, and we hope that this is clearly explained.

All other comments and suggestions of reviewer 2 are extremely constructive and easy to respond to, as we have outlined below.

3. Several conditions including activation of Rab5 cause enlargement of early endosomes. Given this, the connection between PIKfyve inhibition and AD seems tenuous.

A: Yes, several conditions, including activation of Rab5, cause enlargement of early endosomes in neurons. However, very few, if any, conditions that we are aware of cause enlargement of early endosomes, endolysosomal Ca^{2+} accumulation, enlargement and perinuclear clustering of endolysosomes, and autophagic vesicle accumulation as we report in this study with the inhibition of PIKfyve. All of these pathological EAL phenotypes are evident in neurons in the AD brain and many of them are evident in *APOE* ε4 neurons. Therefore, we believe it surely is an important discovery that inhibiting PIKfyve can induce multiple AD pathologies while activation of Rab5 cannot. We thank the reviewer for bringing attention to this, as we may have failed in explaining the significance and novelty of these findings in sufficient detail. As mentioned, we have now included further information in the discussion to clarify this (page 15, line 428-437 and page 18, line 532-537).

4. At the very least, under these conditions, Tau, Abeta peptide levels or other read-outs more directly linked to AD should be evaluated under these conditions. Without these read-outs, the statement “ML-SA1… protects against multiple AD-related EAL neuropathologies (lines 588- 590) isn’t well supported”

A: We respectively disagree that the statement *“ML-SA1… protects against multiple AD- related EAL neuropathologies (lines 588-590) isn’t well supported”*. As we explained in above, ML-SA1 does protect against multiple AD related EAL neuropathologies and we show this for the first time. We agree that measurement of tau and Abeta peptide levels would add further to this study. However, our discovery that PIKfyve inhibition can induce these EAL neuropathologies, which are protected against by TRPML1 activation does stand on its own. Notably, Cataldo and colleagues showed that endocytic pathway abnormalities precede amyloid beta deposition in late onset AD and in Down syndrome (Cataldo AM et al., Amer. J. Pathol. (2000) 157: 277-286).

Reviewer 2 Additional concerns on specific figures:

1. In Fig 1A: The “perinuclear” clustered lysosome authors are examining in AD panels are in fact, lysosomes in glia. This is obvious from morphology of those cells visible even with just LAMP1 staining One can also appreciate their difference from the much larger neuronal cell bodies in Control. A couple of glia visible in this image too. It is more likely the authors have observed the increased astrogliosis in AD tissue. Colocalization with GFAP and iba1 will be needed to distinguish between lysosomal changes in CA1 neurons versus glia in that region.

A: We agree with the reviewer that colocalization of LAMP1 with GFAP and Iba1 is needed to distinguish between lysosomal changes in CA1 neurons versus glia. We respectively disagree that the perinuclear clustering of lysosomes in the AD patients are in fact all lysosomes in glia. The 3D reconstructed cells in Figure 1B were morphologically identifiable as pyramidal neurons, and the perinuclear clustering of lysosomes in AD neurons is highly evident here. Notably, Cataldo *et al.*, report distinct accumulation of cathepsin-D-positive lysosomes in neurons in AD brain sections (Cataldo AM *et al.*, Neuron (1995) 14, 678-80). However, we do agree with the reviewer that the morphology of some of the cells visible with LAMP1 staining in Fig 1A may be glia and thank the reviewer for highlighting this. Thus, we performed double immunofluorescence with LAMP1 and the astrocytic marker GFAP. Our results show that increased levels of LAMP1-positive endolysosomes

© 2023. Published by The Company of Biologists under the terms of the Creative Commons Attribution License (https://creativecommons.org/licenses/by/4.0/).

are observed in the perinuclear regions of GFAP-negative pyramidal neurons in the AD hippocampus. In addition, GFAP-labelled astrocytes with increased levels of LAMP1 immunoreactivity in perinuclear regions were identified in the AD hippocampus (Figure S1A). We were unable to make a definitive conclusion regarding whether Iba-1 labelled microglia also displayed endolysosomal alterations in AD. Together, this data indicates that increases in LAMP1-positive endolysosomes occur in neurons and astrocytes in the AD hippocampus when compared to control hippocampus. We have modified the results (page 6, line 161-166) and discussion text (page 14, line 422-426) accordingly. We thank the reviewer for drawing our attention to this important point.

2. In Figure 1E, F it is unclear what the data points are -mean intensity from 1 neuron? Are all 10 patient samples and controls being looked at here? From the graph and brief description of the statistics, it is unclear what the mean, error bars plotted here are: ideally, mean from multiple neurons per sample should be compared.

A: We apologise that this is unclear. The data presented now in Fig 1D, E is from n = 6 AD cases and n = 8 control cases with 2-5 representative images analysed for each case, together analysing n = 26 control images and n = 26 AD images.

3. Fig 1D: the western blot for LAMP1 normalized with the protein stain is not convincing-one could argue that the protein levels (especially when comparing dark band at 50kDa) is increased in many of the AD samples compared to control (reflected in lack of significance in their quantification).

A: We agree that the western blot (now in Fig 1I, J) shows variable LAMP1 levels when comparing control and AD samples. However, one can see that the total protein stain verifies equality of protein loading of each of the control and AD cases, and we do think the protein stain is a convincing one. These are the levels of LAMP1 present in the control and AD cases. It most likely arises from actual variability in LAMP1 between control and AD cases, although many of the AD cases do have high levels of LAMP1, as pointed out by the reviewer, this did not attain significance due to the variability in LAMP1 levels when comparing the total number of AD and control cases. These results agree with previously published work where western immunoblot analysis also found LAMP1 levels were not significantly different when comparing brain samples from control and AD patients (Bordi *et al*., 2016, Autophagy). These results may also reflect the mixture of cell types that are present in such human brain lysates. We have included additional information in the results (Page 7, lines 191-196) section to clarify this.

4.The observation that LAMP1 immunoreactivity is enriched in neuritic plaques is well established (Condello et al, 2011, Scientific Reports, Nixon et al, 2005 Gowrishankar et al, PNAS, 2015, Kandelepas et al 2016). The results describing this (Line 335) should cite prior work on this observation

A: Yes, it is indeed established that LAMP1 immunoreactivity is enriched in neuritic plaques in the AD brain. We had cited the relevant references in the discussion and now include these in the results section as well (Page 7, line 186-189) and thank the reviewer for pointing out this omission.

Reviewer 3

Advance Summary and Potential Significance to Field

This is a timely manuscript by an excellent group with substantial experience in the calcium and AD fields. The greater lysosomal system is rather under-appreciated in the AD field and therefore this manuscript helps highlight its importance. The writing was excellent, the experiments appear to have been conducted appropriately, and their findings would be an addition to the field. However, I do have some points that once addressed might make this manuscript even stronger.

We thank the reviewer and appreciate their support of our manuscript highlighting the importance of the greater lysosomal system in AD pathogenesis. The comments for the author are extremely

© 2023. Published by The Company of Biologists under the terms of the Creative Commons Attribution License (https://creativecommons.org/licenses/by/4.0/).

valuable and insightful, we have addressed these below, and believe that they have made the manuscript stronger.

Comments for the Author

1.The authors either missed or chose not to include substantial literature showing that ML- SA1 protects against Alzheimer's-like pathology that occurs in other neurological conditions including HIV-1 associated neurocognitive disorder. They might start with the following publication in which it was shown that ML-SA1 promoted calcium efflux, luminal acidification, and cleared both sphingomyelin and Aβ from lysosomes.

Bae, M., Patel, N. Xu, H., Lee, M., Nath, A., Geiger, J.D., Gorospe, M.M., Mattson, M.P., and Haughey, N.J. (2014) Activation of TRPML1 Clears Intraneuronal Aβ in Pre-Clinical Models of HIV-Infection. J. Neurosci. 34 (34): 11485-11503.

A: We thank the reviewer for pointing out this omission. We did mention a related reference to Bae *et al.*, briefly in the discussion (Hui *et al.*, 2019). However, we have now included this information and related references in more detail (Introduction page 5, line 130-133; Discussion page 18, line 543-548). This literature is also important with respect to answering concerns raised in comment 2 below. We have combined this specific literature on HIV-1 associated neurocognitive disorders in the following statement: "Furthermore, ML-SA1 cleared sphingomyelin and Aβ from LAMP1-positive lysosomes in an HIV cell model (Bae et al., 2014) and ML-SA1-induced acidification of endolysosomes blocked LDL-induced increase in intra-neuronal and secreted levels of Aβ (Hui et al., 2019). Hui et al., further showed that antiretroviral drugs increased Aβ levels by de-acidifying endolysosomes and that ML-SA1 prevented the resulting Aβ accumulation (Hui et al., 2021)." We have also included further detail on ML-SA1 protection in other neurological disorders (Discussion page 18, line 541-543).

2.There is also substantial literature on how insults related to AD pathogenesis cause increases in pH of endosomes and lysosomes and how ML-SA1 can rescue not only the pH but also AD-like pathological effects. Therefore, I recommend either measuring vacuolar pH or at the very least addressing these issues in the manuscript. Especially because calcium accumulation, perinuclear clustering, and effects on organellar volume are affected by vacuolar pH.

A: To address the reviewer's concerns we have included information on the substantial literature regarding how insults related to AD pathogenesis, predominantly described in HIV- induced neurocognitive disorders, can cause increases in pH of endosomes and lysosomes and further show that ML-SA1 can rescue not only the pH but also AD-like pathological effects (Discussion page 18, lines 543-548, please see answer to comment 1 above).

To address the reviewer's recommendation, we have provided additional data which measured vacuolar pH in all of the *APOE* iPSC-derived cortical neurons using a dextran conjugated pH-sensitive probe. Our results show that lysosomal pH is not changed in any of the *APOE* isoform expressing cortical neurons (Figure 3C, D). This removes any concerns about possible alterations in vacuolar pH in these cells and shows that *APOE* ε4 iPSC- derived cortical neurons are not de-acidified. It also gives confidence that GPN in these cells is being hydrolysed correctly. We also performed further experiments to measure lysosomal Ca^{2+} *in situ* with Oregon Green BAPTA 5N and Texas Red Dextran as a loading control (Figure 3B). These data further confirm that lysosomal Ca^{2+} is elevated in *APOE* ε4 expressing cortical neurons (Figure 3A, B and related methods (page 22/23, line 684-706) and results (page 9 line 246-255) sections). This further confirms the GPN data and strengthens our findings indicating that lysosomal Ca^{2+} levels are significantly increased in *APOE* ε4 neurons compared to neurons expressing other *APOE* isoforms.

Furthermore, we used BODIPY-pepstatin as an indicator of cathepsin D activity in all the *APOE* neurons. Our results show BODIPY-pepstatin fluorescence is altered in *APOE* e4 neurons, with significantly increased BODIPY-pepstatin total spot fluorescence and area in *APOE* e4 neurons compared to other *APOE* isoforms (Figure S2F-H). There was also a trend towards a decreased number of BODIPY-pepstatin fluorescent spots per cell in the *APOE* e4 neurons, but this was not significant (Figure S2I, methods (page 23, line 708-712), results page 10, line 285-294)). Together,

© 2023. Published by The Company of Biologists under the terms of the Creative Commons Attribution License (https://creativecommons.org/licenses/by/4.0/).

these data indicate that there are increased levels of active cathepsin D in *APOE* ε4 neurons. Notably, increased cathepsin D levels have been described previously in AD neurons (Cataldo AM *et al.*, Neuron 1995: 14, 671-80; Cataldo AM *et al.*, PNAS 1991: 88 10988-1002). This further indicates indirectly that lysosomes are not de-acidified in *APOE* ε4 neurons as if this was the case we would expect to see less and not more active cathepsin D.

3.They were singularly focused on the divalent cation calcium. However, endosomes and lysosomes also contain other divalent cations, the release of which is also influenced by pH and TRPML channels. Therefore, they should at least comment on their possible involvement in the observed subcellular changes as they relate to AD.

A: We have now commented on the possible involvement of other divalent cations, Fe^{2+} and Zn^{2+} contained within endolysosomes that are also influenced by pH and TRMPL1 channels, as they may relate to AD (Results page 10 line 278-284; Discussion page 15 line 451-456). We thank the reviewer for drawing our attention to this as it is important to mention the cation selectivity for TRMPL1 channels which includes Ca^{2+}, Fe^{2+} and Zn^{2+}. Thus, alterations in TRPML1 function in AD could potentially impact release of all three cations from endolysosomes by TRPML1 and their levels within endolysosomes. We focused on Ca^{2+}, as it is the divalent cation found in the lysosome in highest concentration (500 μM, Lloyd-Evans *et al.*, Nat. Med (2008) 14: 1247-55; Christensen Myers and Swanson J.Cell Sci (2002) 1: 599-607) and owing to this excess it is the cation TRPML1 primarily transports at low pH (Li *et al.*, Nat Struct Mol. Biol (2017) 14:1247-55). Furthermore, the data on a physiologically relevant role for TRPML1 in Zn^{2+} and Fe^{2+} transport from endolysosomes is considerably weaker thus rationalising our focus on Ca^{2+}.

Nevertheless, we performed additional experiments with the iPSC-derived *APOE* neurons using the FluoZin3(AM) probe to detect Zn^{2+} accumulation. Our results show that no significant difference in Zn^{2+}levels were detected when comparing the *APOE* isoforms in iPSC-derived cortical neurons (Figure S2A-E; methods (page 23, line 714-719; results text (page 10, line 278-284); discussion page 15, line 451-456). This is not surprising, as Zn^{2+} accumulation in cells with a TRPML1 defect would only be observed when the cells are grown in 100 μM extracellular zinc (Minckley *et al.*, Nat. Commun. 2019: 10, 4806). We did not investigate this, as this concentration of $ZnCl_2$ is toxic to cells.

4.Calcium release experiments were performed with GPN and bafilomycin and showed effects on lysosome calcium levels and release. They could have introduced or commented on possible inter-organellar signaling by the released calcium. They showed changes in relative levels of calcium in lysosomes but not actual levels (probably in the hundreds of micromolar). If they have those data I think they should be included.

A: As discussed in response to Comment 2 above, we performed additional experiments showing increased lysosomal Ca^{2+} *in situ* in *APOE* ε4 cortical neurons compared to other *APOE* isoforms. Furthermore, we measured vacuolar pH in all of the *APOE* iPSC-derived cortical neurons and our results show that lysosomal pH is not changed when comparing *APOE* isoforms in these neurons. These data solidify our original experiments measuring lysosomal calcium levels and calcium release with GPN and bafilomycin. The ionomycin/GPN experiment rules out ER stores (Smith JB, Zheng and Lyu, Cell Calcium 1989: 10, 125-134) contributing to increased endolysosomal calcium levels in the *APOE* ε4 neurons. We have commented on this in the results text (Page 9, line 246-255).

We thank the reviewer for drawing our attention to commenting on the possible inter- organellar signalling by the released calcium. We had done this is part in the previous discussion but emphasise this further in the revised manuscript (Discussion page 16, line 468- 469).

5.Phosphoinositides are very labile substances. Others and they could not measure levels of the major endogenous agonist of TRPML channels PI(3,5)P2. It might be helpful to know what the postmortem intervals for the human brain samples were and whether there was any correlation between PI levels and post mortem intervals.

© 2023. Published by The Company of Biologists under the terms of the Creative Commons Attribution License (https://creativecommons.org/licenses/by/4.0/).

A: The post-mortem intervals for the brain tissue analysed for phosphoinositide levels is included in Table 1. We did not find any correlation between PI levels and post-mortem interval in these samples and now mention this in the Results text (page 7, line 206-207). The inability to measure PI(3,5)P2 levels by current mass-spectrometry technology is due to the fact that this PI species is present at very low levels but also because the technology does not yet allow this specific stereoisomer to be distinguished from the PI(4,5)P2 and PI(3,4)P2.

There are several approaches being pursued in attempts to enable this. We mention this in the Discussion (page 17, line 521-525).

Minor issues:

In the Results section they need to refer to their data as 'these data' not "this data".
A: This has been changed throughout.

Reviewer 4

Advance Summary and Potential Significance to Field:

The authors present a thorough evaluation of the contribution of TRPML1 to LOAD phenotype in patient brain sections, APOE iPSC-neurons and rat primary neurons. They show that in LOAD post-mortem CA1/CA3 brain sections that LAMP1+ vesicles cluster in perinuclear endolysosomes (EL), APOE4 iPSC cortical neurons have decreased TRPML1- induced EL Ca2+ release compared to other APOE allelic isoforms and pharmacological inhibition of TRPML1 activation via PIKfyve-PIP3 recapitulated LOAD phenotype in primary neurons. This phenotype was rescued using ML-SA1 agonist. Confirming the mechanistic link between PIKfyve-PI(3,5)P2-TRPML1 in LOAD neurons has not been done before which makes this manuscript novel and provides significant new information to the field relating EL vesicular transport and more specifically TRPML1 to Alzheimer's disease pathobiology.

We thank the reviewer for highlighting the novelty of our study providing new information on EL vesicular transport and TRPML1 in Alzheimer's disease. The comments for the author are extremely valuable and we have addressed these important points below.

Comments for the Author:

1.Overall, the manuscript is very well written apart from the continued fact that the figures and sub-figure orders do not follow the chronology of the Results body text. This culminates in the final two Results sections flipping back and forth between Figures 5 and 6. I think this is easily resolved and needs to be altered.
A: We thank the reviewer for pointing this out. As mentioned by the reviewer, this was relatively easy to resolve. Thus, we have made alterations so that the figures and sub-figure order follow the chronology of the results body and the results text no longer necessitates flipping back and forth between Figures 5 and 6 (now Figures 6 and 7).

Finally, I see no reason why all the data referred to as "data not shown" should not be shown, some of it is important in terms of reference or substantiating other datasets. I would recommend much of it is presented in Supplementary Data.

We have now presented the "data not shown" in Figure 4C and the Supplementary Data (Figure S1 and S3).

Major Comments
1.ICC is conducted using LAMP1 in brain sections and iPSC-neurons but Rab7 in rat primary neurons to label EL. It could be argued that neither are bona fide markers of EL, with LAMP1 being a mature lysosome marker and Rab7 being implicated in many aspects of membrane trafficking in the late endocytic and autophagic network. This may require more specific classifications. This comment also cross-references to comment 6.

© 2023. Published by The Company of Biologists under the terms of the Creative Commons Attribution License (https://creativecommons.org/licenses/by/4.0/).

A: We agree with the reviewer and appreciate that the use of LAMP1 in human brain sections but Rab7 in rat primary neurons should be considered. The LAMP1 antibody we used predominantly in the brain sections is specific for human/monkey. We evaluated different LAMP1 antibodies in the rat primary neurons (see response to comment 6 below), but neither gave vesicular staining, whereas Rab7 clearly labelled endolysosomes. Likewise, anti-human Rab7 antibody did not give above background immunofluorescence in human brain sections.
We take the reviewer's comment on board as well to define more specific classifications regarding LAMP1 and Rab7 as *bona fide* markers of endolysosomes by commenting on this in the discussion (page 16, line 476-484). Both LAMP1 and Rab7 are used most commonly in the literature to label endolysosomes and as TRPML1's functions are linked to the transition from late endosome to lysosome, both endolysosomal markers can be considered appropriate. However, we concur that ideally the same endolysosomal marker should be used in the different cell systems.

2.It is disappointing that PI(3,5)P2 cannot be detected on mass spec, somewhat diminishing the link between PI(3,5)P2 and TRPML1. Mass spec analyses of phosphoinositol species in LOAD patients compared to age-matched controls shows no significant difference in PI or total PIP and a significant increase in PIP2 and PIP3. However, only the increase in PIP2 is discussed in terms of relevance. This requires an expanded justification in the Results section.

A: It is indeed a disappointment that PI(3,5)P2 cannot be detected yet by mass-spectrometry approaches that measure phosphoinositol species. This is because PI(3,5)P2 levels are of extremely low abundance and the mass-spectrometry methodology cannot, as yet, distinguish between this and related PIP2 regioisomers, although very recent research approaches indicates this may be possible. We have added the following information regarding this to the discussion (page 17, line 521-525): "Very recent research suggests independent measurement of PIP2 regioisomers, enabling measurement of PI(3,5)P2 (Morioka *et al.* 2022). Achieving this should enable further understanding of mechanisms by which the dynamics of this vital low abundance phosphoinositide couples late endolysosomal trafficking and function to TRPML1 activation in health and neurodegenerative disease". We agree with the reviewer that the increase in PIP3 in AD cases compared to controls requires an expanded justification in the results sections. We have now included this is the results (page 8, line 212-215) stating: "PIP3, whose levels we show to be increased in the AD group, is the major activator of Akt, a kinase that is known to be hyper-activated in AD neurons (Griffin et al., 2005; Moloney et al., 2010) and which is a primary regulator of EAL dynamics via the mTOR signalling axis (Boland et al., 2018)."

3.The data showing that APOE4 EL load elevated Ca2+ and that release is compromised through pH-dependent TRPML1 activity is compelling and well presented in iPSC-derived neurons

A: We thank the reviewer for these favourable comments on our findings of compromised TRPML1 calcium activity and elevated Ca^{2+} in *APOE ε4* expressing iPSC-derived neurons. We have now provided further detailed analysis using *in situ* probes to measure lysosomal Ca^{2+} and showing that there is no alteration in vacuolar pH in *APOE* ε4 neurons in response to reviewer 3. These data (Figure 3 and S2 and related results text) add further strength to our original findings.

4.The ability of ML-SA1 to rectify TRPML1 activity following PIKfyve inhibition in rat cortical neurons is equally compelling.

A: We further thank the reviewer for recognising that these data are compelling. Our new findings that ML-SA1 rectifies TRMPL1 activity following PIKfyve inhibition in rat cortical neurons agree with previous findings showing activation of TRPML1 by ML-SA1 in fibroblasts and DRG neurons rescues cellular phenotypes in FIG4 mutant cells, which cannot produce PI(3,5)P2 (Zou J et al., J. Neurosci., 35(17): 6801-12). Together, these findings point to the potential of targeting TRPML1 therapeutically in neurodegenerative disease.

5.The time and dose-responsive vacuolisation caused by YM201636 treatment in rat primary neurons is clear with Rab7. As the authors describe this is not seen with Rab5, EEA1 and LBPA I think it would be equally important to show that it also occurs with LAMP1 even if this is only conducted at 24h with 4μM of YM201636. This would provide consistency in vacuolisation between

© 2023. Published by The Company of Biologists under the terms of the Creative Commons Attribution License (https://creativecommons.org/licenses/by/4.0/).

the different cells analysed in the manuscript.

A: We agree with the reviewer that it is important to also show LAMP1 vacuolisation caused by YM201636 treatment in rat primary neurons, as this would provide consistency between the different cells analysed in the human brain and in rat primary cortical neurons. As mentioned in the response to comment 1 above, we performed a number of experiments in attempts to achieve this. With regard to the reviewers specific request to show LAMP1 localisation to vacuoles produced by YM201636, we tested multiple different LAMP1 antibodies on rat primary cortical neurons, which included those from Abcam (Clone H4A3), and Developmental Studies Hybridoma Bank (DSHB, clone 1DB4). Unfortunately, we were unable to obtain a quality of immunolabelling in rat brain primary neurons with LAMP1 that provided sufficient labelling of endolysosomal compartments achieved in human tissue. We have now addressed this in the Discussion (page 16, line 474-476). Here we state that these results describing vacuoles in endolysosomal compartments in the AD brain and in rat primary neurons are not directly comparable due to the use of different endolysosomal markers in human brain and in rat primary neurons (page 16, line 476-484).

Minor Comments

1. *Figure 1A-C. There is no evidence presented that these are neurons, the staining is LAMP1 ICC and DAPI nuclear stain.*
2. *Figure 1I & J. There is no evidence presented that the stained cells are pyramidal neurons as described in the text. The staining is LAMP1 (red), PHF1 (green) ICC and DAPI nuclear stain.*

A: We agree with the reviewer that there was no definitive evidence presented that the LAMP1 immunofluorescence and endolysosomal pathology from the Alzheimer's disease sections is within neurons, albeit some cells show a pyramidal neuronal morphology. We performed double immunofluorescence with LAMP1 and the astrocytic marker GFAP. Here, our results show that increased levels of LAMP1-positive endolysosomes are observed in the perinuclear regions of non-GFAP immunoreactive pyramidal neurons and GFAP-labelled astrocytes with increased levels of LAMP1 immunoreactivity in perinuclear regions in the AD hippocampus (Figure S1A). We were unable to make a definitive conclusion regarding whether Iba-1 labelled microglia also displayed endolysosomal alterations in AD. Together this data indicates that increases in LAMP1-positive endolysosomes occur in neurons and astrocytes in the AD hippocampus when compared to control hippocampus. We have modified the results (page 6, line 153-171) and discussion text accordingly (page 14, line 422-426).

3. *Figure 1D shows a western blot for LAMP1 on Braak staged tissue but is mentioned much later in the text then quantified in Fig. 1H. It would make sense to me having the figures in order of textual description.*

A: Figure 1 is now in order of textual description.

4. *Figure 3A should be sub-categorised into (i) and (ii).*

A: Fig 3A is now sub-categorised.

3C should precede 3B in my mind as these are some of the data shown in B. Why not show all the traces i.e. with and without inhibitor (which is described as GW405833 in the body text but ML-SI1 in the figure.

A: This now also done. All traces are now shown in Figure 4A and the order is as recommended by the reviewer. We thank the reviewer for pointing out the discrepancy in referring to the used TRPML1 antagonist sometimes as ML-SI1 and sometimes as GW405833. Indeed, GW405833 is a very close analogue to ML-SI1, both inhibit TRPML1 and GW405833 is often referred to as ML-SI1 (even on the supplier page). We have corrected this now throughout the whole manuscript and call it GW405833.

5. *Fig. 3. The data for positive activation of TRPML1 with ML-SA1 should be presented, even if it is included as Supplementary Data.*

© 2023. Published by The Company of Biologists under the terms of the Creative Commons Attribution License (https://creativecommons.org/licenses/by/4.0/).

A: This data is now presented in Figure 4C. This analysis was also repeated and more detail regarding this is given in results text (page 9/10, line 270-277).
6. Fig. 3. It would be good to see the Ca spark Fura-2 profiles with as well as without TRPML1 inhibitor.

A: This is now included as requested in comment 4 (Figure 4A).

7. Fig. 4. The data for YM201636 inhibition of PIKfyve at 6h should be presented in Supp Data. Conversely, data in Fig. 4C could go into supps as 4B shows the reversing effect of ML-SA1.

A: We thank the reviewer for this suggestion, but politely disagree. We think the data for 6h and 24h is presented better together showing a further increase in the size of Rab7-positive endolysosomes (now Fig 6A). Furthermore, Figure 4C (now Fig 5C) is different from Figure 4B (now Fig 5B) and important on its own showing that ML-SA1 by itself does not deplete/lower the lysosomal Ca^{2+} store at 24h, although it does rescue YM201636-caused Ca^{2+} accumulation.

8. Fig. 5. Rab5, EEA1 and LBPA ICC should be presented in Supps. It is relevant to show proof that this effect is seen specifically with Rab7.

A: These data are all now presented in the Supplementary Data (Figure S3).

Second decision letter

MS ID#: JOCES/2022/259875

MS TITLE: The synthetic TRPML1 agonist, ML-SA1, rescues Alzheimer-related alterations of the endosomal-autophagic-lysosomal system

AUTHORS: Aleksandra Somogyi, Emily D. Kirkham, Emyr Lloyd-Evans, Jincy Winston, Nicholas D. Allen, John Mackrill, Karen E. Anderson, Phillip Thomas Hawkins, Sian E. Gardiner, Helen Waller-Evans, Rebecca Sims, Barry Boland, and Cora O'Neill
ARTICLE TYPE: Research Article

We have now reached a decision on the above manuscript.

To see the reviewers' reports and a copy of this decision letter, please go to: https://submit-jcs.biologists.org and click on the 'Manuscripts with Decisions' queue in the Author Area. (Corresponding author only has access to reviews.)

As you will see, the reviewers gave favourable reports but raised one last critical point (besides careful editing of the manuscript) that will require amendments. I hope that you will be able to carry these out because I would like to be able to accept your paper, depending on further comments from reviewers.

Please ensure that you clearly highlight all changes made in the revised manuscript. Please avoid using 'Tracked changes' in Word files as these are lost in PDF conversion.

I should be grateful if you would also provide a point-by-point response detailing how you have dealt with the points raised by the reviewers in the 'Response to Reviewers' box. Please attend to all of the reviewers' comments. If you do not agree with any of their criticisms or suggestions please explain clearly why this is so.

Reviewer 1

Advance summary and potential significance to field

© 2023. Published by The Company of Biologists under the terms of the Creative Commons Attribution License (https://creativecommons.org/licenses/by/4.0/).

The manuscript by Somogyi and colleagues describes the supportive experimental evidence for the potential involvement of TRPML1 dysfunction in the pathogenesis of AD. Key findings were obtained from human iPSC-derived neurons expressing ApoE4 genes (risk factor for late onset AD), which were shown to have reduced TRPML1-associated Ca2+ release. Their hypothesis was also tested in primary neurons using pharmacological tools (e.g. depletion of PiP2 vua PIKfive inhibition. The role of TRPML1 dysfunction in the EAL abnormalities in AD is an interesting possibility. Although presented data appear consistent with the hypothesis further studies may be necessary to prove it right. I have one technical reservation regarding the Ca2+ only explanation. Please see below.

Comments for the author

My main question is whether using Ca2+ and Zn2+ fluorescence probes described in the paper can disprove the role of zinc in TRPML-1-induced fluorescence in this study, especially because TRPML1 channels are permeable to both. In one of early papers describing Fura-2 from Roger Tsien's lab (Grynkiewicz et al., JBC 6: 3440-3450, 1985), they briefly mentioned that Zn2+ has not only higher affinity to FUra2 than Ca2+, but also exhibits the identical fluroescence changes as Ca2+. Hence, without using TPEN (cell-permeant zinc chelator), one cannot know whether the altered Fura-2 signal is from Ca2+ or Zn2+ (Martin et al., Cell Calcium 40:393-402, 2006). In addition, based on one report, Oregon Green 488 BAPTA-1 may stain metals such as zinc! (Figueroa et al, Metallomics, 2014). I want to know how much of the calcium signals reported in the paper are TPEN-quenchable.

Second revision

<u>Author response to reviewers' comments</u>

Response to Reviewers

We thank the reviewers for their review and for their insightful comments. We have thoughtfully addressed them in this response and appropriate revisions have been made in the manuscript.

Reviewer 1

Advance Summary and Potential Significance to Field:

The manuscript by Somogyi and colleagues describes the supportive experimental evidence for the potential involvement of TRPML1 dysfunction in the pathogenesis of AD. Key findings were obtained from human iPSC-derived neurons expressing ApoE4 genes (risk factor for late onset AD), which were shown to have reduced TRPML1-associated Ca2+ release. Their hypothesis was also tested in primary neurons using pharmacological tools (e.g. depletion of PiP2 via PIKfive inhibition. The role of TRPML1 dysfunction in the EAL abnormalities in AD is an interesting possibility. Although presented data appear consistent with the hypothesis, further studies may be necessary to prove it right. I have one technical reservation regarding the Ca2+ only explanation. Please see below.

We thank the reviewer for their review, and appreciate their support of our manuscript highlighting the potential involvement of TRPML1 dysfunction in the pathogenesis of Alzheimer's disease. We have addressed the one technical reservation that the reviewer has, by responding to the reviewer's main question below. We hope that our detailed response allays the reservation of the reviewer that Zn^{2+} release is contributing to the TRPML1-induced fluorophore signal in our study. We can provide additional experimental data, that further supports our response, in confidence, if required.

Comments for the Author:

My main question is whether using Ca2+ and Zn2+ fluorescence probes described in the paper can disprove the role of zinc in TRPML-1-induced fluorescence in this study, especially because TRPML1 channels are permeable to both. In one of early papers describing Fura-2 from Roger

© 2023. Published by The Company of Biologists under the terms of the Creative Commons Attribution License (https://creativecommons.org/licenses/by/4.0/).

Tsien's lab (Grynkiewicz et al., JBC 6: 3440-3450, 1985), they briefly mentioned that Zn2+ has not only higher affinity to Fura2 than Ca2+, but also exhibits the identical fluroescence changes as Ca2+. Hence, without using TPEN (cell-permeant zinc chelator), one cannot know whether the altered Fura-2 signal is from Ca2+ or Zn2+ (Martin et al., Cell Calcium, 40:393-402, 2006). In addition, based on one report, Oregon Green 488 BAPTA-1 may stain metals such as zinc! (Figueroa et al, Metallomics, 2014). I want to know how much of the calcium signals reported in the paper are TPEN-quenchable.

A: We acknowledge the reviewer's concern regarding a potential theoretical role of Zn2+ in the TRPML1-induced fluorescence observed in this study. In our previous response, we have highlighted reasons why it is very unlikely that TRPML1-induced fluorescence in our study could be driven by Zn^{2+} as well as Ca^{2+}. To address this in further detail we have responded to each question that is raised by the reviewer within their main question above, albeit there is some overlap between these questions.

1. My main question is whether using Ca2+ and Zn2+ fluorescence probes described in the paper can disprove the role of zinc in TRPML-1-induced fluorescence in this study, especially because TRPML1 channels are permeable to both.
The fluorescence probe, Fura-2, used in our paper will detect both Ca^{2+} and Zn^{2+}, and thus, as the reviewer states, it is theoretically possible that Fura-2 may detect Zn^{2+} that could potentially be released by TRPML1 channel activation, however, this is highly unlikely to occur in a cellular context (see further detailed discussion on this below). The reviewer states that TRPML1 channels are permeable to both Ca^{2+} and Zn^{2+}. However, we would like to point out that the original publication that characterized TRPML1 ion channel permeability (Dong *et al.*, Nature, 2008, 455: 992-6) showed the channel was permeable to Ca^{2+}, Fe^{2+} and also Zn^{2+}, Mn^{2+} and most other divalent trace metals. This paper by Dong et al., analysed TRPML1 using the activating V424P 'varitint waddler' (Va) mutation, as was often the case in TRPML studies, prior to the discovery of endogenous/small molecule activators and inhibitors. Notably, the original Va mutation, which occurred spontaneously in TRPML3, disrupts the transmembrane domain-5 alpha helix and changes fundamental properties of the channel pore (Kim et al., 20110, 285:16513-20). Importantly, this includes loss of Ca^{2+} ion selectivity. It thus remains entirely possible that the TRPML1 permeability for Zn^{2+} in that study was due to an increased pore size resulting from the Va mutation, in particular in the absence of electrophysiology data on wild-type TRPML1 which may confirm this observation.

2.In one of early papers describing Fura-2 from Roger Tsien's lab (Grynkiewicz et al., JBC 6: 3440- 3450, 1985), they briefly mentioned that Zn2+ has not only higher affinity to Fura2 than Ca2+, but also exhibits the identical fluroescence changes as Ca2+. Hence, without using TPEN (cell-permeant zinc chelator), one cannot know whether the altered Fura-2 signal is from Ca2+ or Zn2+ (Martin et al., Cell Calcium, 40:393-402, 2006)

As the reviewer states Fura-2 has a high affinity for both Ca^{2+} and Zn^{2+} and therefore theoretically Fura-2 could detect both Ca^{2+} and Zn^{2+} release from the TRPML1 channel, and this is an important consideration theoretically. The paper cited by the reviewer (Martin *et al.*, 2006, Cell Calcium 40: 393-402) investigated the dynamic range of Fura-2 i.e. the extent by which the Fura-2 fluorescence changes as Ca^{2+} and Zn^{2+} concentrations change. However, in the cellular context the concentration achieved by Zn^{2+} in the paper by Martin et al., would never be achieved.

To elaborate further, in the cellular context, Ca^{2+} concentration is in the range of 10-100 nM in the cytoplasm at rest and rises to around 1-10 µM following cellular activation and is therefore fully in the dynamic range of Fura-2 described in the citation of Martin et al. In contrast, Zn^{2+} concentrations within the cell are believed to change by only 1 nM following activation (Krezel and Maret, J Biol. Inorg. Chem. 2006, 11: 1049-62). Furthermore, total cellular free Zn^{2+} is believed to be in the pM range (Bozym *et al.*, Exp Biol. Med 2010, 235: 741-50; Bozym *et al.*, ACS Chem Biol., 2006, 17: 103-11), which is at least one order of magnitude lower than free Ca^{2+}. Thus, as shown by Krezel and Maret, 2006, Fura-2 is unable to respond to such small changes in Zn^{2+} concentrations. These authors, and others, further indicate that cellular Zn^{2+} concentrations will never change by as much as 1 µM, and such changes are believed to be extremely cytotoxic to cells (Bozym *et al.*, 2010, Exp Biol. Med. 235:741-50).

© 2023. Published by The Company of Biologists under the terms of the Creative Commons Attribution License (https://creativecommons.org/licenses/by/4.0/).

In summary, a high experimental KD of Fura-2 for Zn^{2+} in theoretical experimental conditions as described by Martin et al., is meaningless in the cellular context, when the concentration of Zn^{2+} inside a cell is so much lower in comparison to Ca^{2+}, and where Fura-2 will respond to Ca^{2+} and not Zn^{2+}. We would like to further mention information from our response to the previous review that data showing zinc accumulation in TRPML1 deficient MLIV patient cells is only observed when the cells are grown in extracellular medium supplemented with 100 μM Zn^{2+} (as outlined in many publications by Kirill Kiselyov's team for example), which is non- physiological.

Unfortunately, employing TPEN would not enable the conclusion that the Fura-2 signal could be due to Zn^{2+} release via the TRPML1 channel in our studies. This is because TPEN is itself not particularly specific and also chelates Ca^{2+} as shown by Morgan *et al.,* (Cell Calcium, 2012, 52: 481-487) where TPEN was shown to reduce NAADP-mediated Ca^{2+} release from lysosomes. Importantly, NAADP does not release Zn^{2+} ions, indicating TPEN is binding Ca^{2+} and other studies have independently shown TPEN chelates Ca^{2+} (e.g. Shumaker *et al.,* Cell Calcium 98, 23: 151-64). Together, these findings indicate that the result of an experiment employing TPEN will be uninterpretable. We have now inserted some detail in our manuscript to address these concerns of the reviewer (page 10, line 283-289), regarding, TPEN and also the potential of Fura-2 to detect Zn^{2+} release from TRPML1 where we reiterate that this is highly unlikely in the cellular context of our work. We thank the reviewer for drawing our attention to this.

In addition, based on one report, Oregon Green 488 BAPTA-1 may stain metals such as zinc! (Figueroa et al, Metallomics, 2014). I want to know how much of the calcium signals reported in the paper are TPEN-quenchable.

The one report cited by the reviewer to support the binding of Oregon Green 488 BAPTA-1 (OGB) to Zn^{2+} (Landero-Figueroa et al., Metallomics, 2014, 6: 301-15) is not a typical ion imaging study. Rather, it is an ICP-MS heavy metal study coupled to HPLC to somewhat interpret the fluorophore readings. Moreover, and of concern, the work describes in its method section that the dyes were loaded at 37°C for 30 min. This is an extremely uncommon practice in the field, since it creates the risk that the dyes will compartmentalize into organelles rather than load just the cytoplasm (described in detail in Thomas *et al.*, Cell Calcium, 2000, 28: 213- 223), resulting in a potential artefact. Furthermore, this one report does not show any images of cells, as all the work is based on HPLC. We question the relevance of this report to our study, where to avoid this pitfall, we loaded all Ca^{2+} dyes in our study at room temperature and it is clearly visible in our cellular imaging data that we are measuring cytoplasmic Ca^{2+}.

Finally, we would like to inform the editor and reviewer that we actually have data that can prove that Zn^{2+} has no effect to Fura-2's ability to measure Ca^{2+}. These data are critical for another study and therefore cannot be included in this manuscript and are shared here in confidence, the details of our findings are outlined below.

We are investigating NPC1 protein (NPC intracellular cholesterol transporter 1) cell function to understand NPC1 (Niemann-Pick disease type C1), a major lysosomal storage disorder, caused by mutations in the *NPC1* gene. In our study, we show that lysosomal mutant NPC1 is a high affinity, low capacity lysosomal Zn^{2+} transporter, where Zn^{2+} accumulates in lysosomes (without the need to incubate cells in extremely high non-physiological 100 μM $ZnCl_2$, as has been done in any study examining MLIV (TRPML1) mutant cells). Importantly, our study shows that TRPML1 activation via ML-SA1 in NPC1 mutant fibroblasts increases Fura-2 levels but does not mobilise the Zn^{2+} in these cells, as there is no alteration in lysosomal Zn^{2+} concentrations measured by Fluozin, yet there is a measurable Fura-2 mobilisation of Ca^{2+} release.

Furthermore, our previous work showed that cells expressing the NPC1 mutant have lower levels of lysosomal Ca^{2+} than non-mutant NPC1 cells, when Ca^{2+} is measured *in situ* or by bursting lysosomes using GPN (Lloyd-Evans *et al.*, Nat. Med., 2008, 14: 1247-55). As GPN will also release intra-lysosomal Zn^{2+}, one would anticipate increased Fura-2 levels in these NPC-1 mutant cells if Fura-2 was also able to detect this Zn^{2+} release. However, even in the context of higher levels of

© 2023. Published by The Company of Biologists under the terms of the Creative Commons Attribution License (https://creativecommons.org/licenses/by/4.0/).

intra-lysosomal Zn^{2+} and lower Ca^{2+}, Fura-2 prioritises Ca^{2+} due to its relatively higher concentration than Zn^{2+}. Together our data clearly support our conclusion Fura-2 is measuring Ca^{2+} and not Zn^{2+} in the cellular context of the experiments performed in our study. These experimental data can be shown to the editor in a confidential manner if requested.

Reviewer 3

Advance Summary and Potential Significance to Field

I think the authors responded well to the comments made by the reviewers.

Comments for the Author:
There remained a few issues with grammar but otherwise no additional changes recommended.

A: We thank the reviewer and appreciate their support of our manuscript. We have carefully proofread and corrected the few remaining grammatical issues. Minor grammar changes were not highlighted in the text.

Third decision letter

MS ID#: JOCES/2022/259875

MS TITLE: The synthetic TRPML1 agonist, ML-SA1, rescues Alzheimer-related alterations of the endosomal-autophagic-lysosomal system

AUTHORS: Aleksandra Somogyi, Emily D. Kirkham, Emyr Lloyd-Evans, Jincy Winston, Nicholas D. Allen, John Mackrill, Karen E. Anderson, Phillip Thomas Hawkins, Sian E. Gardiner, Helen Waller-Evans, Rebecca Sims, Barry Boland, and Cora O'Neill
ARTICLE TYPE: Research Article

Thank you for your comments on the technical concerns of Reviewer 1. I am happy to tell you that your manuscript has been accepted for publication in Journal of Cell Science, pending standard ethics checks.

© 2023. Published by The Company of Biologists under the terms of the Creative Commons Attribution License (https://creativecommons.org/licenses/by/4.0/).
